# An Interval Belief Rule Base Method with Attention Enhancement for Bearing Fault Diagnosis Under Variable Operating Conditions

**DOI:** 10.3390/s26030891

**Published:** 2026-01-29

**Authors:** Bing Chen, Jingying Li, Hongyu Li

**Affiliations:** School of Computer Science and Information Engineering, Harbin Normal University, Harbin 150025, China; 2024300700@stu.hrbnu.edu.cn (B.C.); 2024300713@stu.hrbnu.edu.cn (J.L.)

**Keywords:** interval belief rule base, rolling bearing fault diagnosis, cross-condition diagnosis, generalization ability

## Abstract

As bearings are critical mechanical components, their actual operating conditions exhibit notable dynamic complexity. Multiple factors—including rotational speed fluctuations, sudden load changes, and environmental disturbances—interact in a strongly coupled fashion. This imposes severe challenges on traditional fault diagnosis methods, such as limited interpretability, weak adaptive capacity, and elevated misjudgment rates. Therefore, this paper proposes an Interval Belief Rule Base model integrated with an attention mechanism (IBRB-a) under variable operating conditions. The proposed model combines expert knowledge’s ability to quantify uncertainty with a data-driven adaptation mechanism, thereby addressing the challenge of variable operating conditions in complex industrial systems. First, a novel interval rule construction method is incorporated into the traditional IBRB model, and kernel density estimation (KDE) is employed to select reference values. Second, during the model reasoning process, a two-stage fusion strategy based on Evidential Reasoning (ER) is adopted: progressive information fusion is implemented via the ER analysis algorithm and the ER rule algorithm, which effectively mitigates the interval uncertainty under variable operating conditions. Finally, the constrained projected covariance matrix adaptive evolution strategy (P-CMA-ES) is employed to optimize the model. Furthermore, experimental validation under variable operating conditions is conducted via Case Western Reserve University and Southeast University bearing datasets. The effectiveness and generalizability of the proposed method are validated by the experimental result.

## 1. Introduction

Bearing is one of the key rotating components of large-scale mechanical equipment [1,2,3]. Effective bearings fault diagnosis can avoid significant social and economic losses [4,5]. Therefore, the fault diagnosis of rolling bearing is of great significance in avoiding accidents, reducing the maintenance cost of machinery.

Presently, fault diagnosis methods can be classified into three main categories: knowledge-driven methods [6,7,8,9], data-driven methods [10,11,12,13,14,15,16,17], and hybrid-driven methods [18,19,20,21,22]:

(1) Knowledge-driven methods [23]. By leveraging expert experience and domain knowledge, these methods can effectively diagnose faults in systems: they make decisions via rules and reasoning to identify and resolve faults.

(2) Data-driven methods [24,25,26]. By utilizing historical data and real-time sensor data, these methods can effectively identify and predict system faults, while demonstrating notable advantages with regard to adaptability and automation level. However, traditional methods often face the challenge of “catastrophic forgetting” when addressing dynamically emerging fault categories. To this end, lifelong learning paradigms aimed at achieving continuous learning have attracted significant attention. For instance, a generalized lifelong learning framework based on an inverter has been proposed to balance model stability and plasticity [27]. Furthermore, targeting specific diagnostic scenarios such as low-speed bearings, this framework has been further refined and applied, with mechanisms such as dynamic learnable pruning introduced to enhance adaptability to incremental information regarding weak faults [28]. These works provide novel insights for the online updating and upgrading of data-driven models without the need for retraining.

(3) Hybrid-driven models [29,30]. By combining the combination of the advantages of data-driven and knowledge-driven approaches, hybrid-driven models either constrain data learning via physical models or optimize the parameters of knowledge models via data. The combination of these two approaches can enhance the generalizability and engineering applicability of the models.

Under variable operating conditions, bearing fault diagnosis faces significant challenges. While knowledge-based methods can leverage expert experience to address certain complex scenarios, they fundamentally rely on a fixed rule system. When confronted with frequent changes in operating conditions, their original rule base fails to rapidly incorporate fault patterns associated with new operating conditions—this leads to a disconnect between diagnostic rules and actual fault mechanisms, as well as a significant decline in adaptability. In contrast, data-driven methods focus on the laws governing data distribution and establish fault mapping models through large-scale sample training. However, variable operating conditions induce significant shifts in the data distribution, which in turn impairs the models’ generalizability. Furthermore, most data-driven models feature a “black-box” structure, resulting in poor interpretability. In contrast, hybrid-driven methods deeply integrate expert knowledge with data to construct a diagnostic framework more suited to variable operating conditions. On the one hand, they use knowledge-driven methods to decouple relationships from a physical perspective, thereby reducing reliance on large-scale labeled data. On the other hand, they employ data-driven approaches to optimize parameters in real-time, acclimate to operating condition fluctuations, and detect subtle fault features. Through this integration, the framework not only ensures the interpretability of diagnostic logic but also enhances the model’s adaptability, making it a promising approach for addressing the challenges posed by complex operating conditions.

Although the interval belief rule base (IBRB) has achieved some progress, it still has limitations when addressing bearing fault diagnosis in variable operating conditions. From a theoretical perspective, the core challenge of this problem lies in the mismatch between the data distribution and feature patterns. During equipment operation, operating condition parameters change dynamically according to production requirements, which directly alters bearing-related data, resulting in a nonlinear mapping relationship between fault features under different operating conditions. Moreover, random fluctuations in environmental factors introduce substantial non-stationary noise. This further degrades the signal-to-noise ratio (SNR) of the features, leading to feature ambiguity due to multi-source interference and significantly increasing the computational burden of the prediction process. Therefore, the specific problems faced are as follows:

(1) Traditional data-based sampling methods often fail to capture key feature regions accurately—due to their reliance on preset distribution assumptions, which makes it difficult to balance model efficiency and interpretability.

(2) Rules constructed under specific operating conditions fail to cover new operating scenarios, resulting in coverage gaps within the rule base. Furthermore, the “dynamic optimal solution drift” issue encountered during the parameter optimization process hinders the model from converging to a stable optimal solution. Ultimately, this approach fails to meet the requirements for interpretability and adaptability in industrial applications.

To overcome the limitations of traditional IBRB under varying operating conditions, this study introduces an attention mechanism as a core dynamic adjustment component. This design is grounded in a dual rationale. Theoretically, through dynamic re-weighting, attention enables the model to adaptively screen critical features and activate corresponding rules based on the current input. This transforms the fixed knowledge base into an inference system that flexibly adjusts to concept drift, directly addressing the challenge of “dynamic optimal solution drift.” Practically, the attention weights embedded within the interpretable rule structure possess clear physical meanings, rendering the focus of each decision traceable and explainable. Thus, the integration of the attention mechanism is not a mere augmentation but a deliberate design choice aimed at simultaneously enhancing the model’s adaptability to varying conditions and the transparency of the decision-making process, thereby meeting the dual industrial diagnostic requirements of robustness and credibility.

This is essentially different from existing methods: (1) Distinction from existing IBRB variants: Most mainstream studies focus on improving optimization algorithms to pursue static optimal parameters, while the IBRB-a introduces the attention mechanism as a dynamic modulator, enabling the model to adaptively adjust rule confidence based on current input features. This fundamentally alleviates the problem of “dynamic optimal solution drift”, transforming the optimization objective from searching for a fixed point to learning a conditionally optimal parameter space. (2) Distinction from conventional attention hybrid models: In the “deep learning + attention” framework, the attention module is usually a black box. In contrast, the IBRB-a explicitly embeds attention weights into the belief rule structure, with a clear physical meaning—it directly represents the support degree of different features for a specific rule under the current operating condition. Thus, while enhancing adaptive capability, the model strictly maintains interpretability. Therefore, the main contributions of this paper are as follows:

(1) A reference point selection approach based on kernel density estimation (KDE) is proposed. By examining the local density features of the data distribution, this method dynamically refines the sampling strategy and independently identifies key feature regions. This enhances the model’s operational efficiency and effectively safeguards the interpretability of the algorithm.

(2) An enhanced projection covariance matrix adaptation evolution strategy (P-CMA-ES) is utilized to optimize the model by integrating an attention mechanism with a dynamic step-size adjustment mechanism. This algorithm applies attention weights to impose interpretability constraints on the diagnostic decision-making process and accommodates dynamic variations in operating conditions through adaptive step-size adjustment, thereby markedly improving the model’s interpretability and applicability in industrial settings.

The structure of this paper is as follows: Section 2 defines the research problem and presents an overview of the IBRB-a model; Section 3 details the reasoning mechanisms and optimization procedures of the proposed model; Section 4 offers experimental validation results; and Section 5 draws conclusions and explores directions for future research.

## 2. Problem Description and Introduction to the IBRB-a Method

This section discusses the limitations of the IBRB method for bearing faults diagnosis under variable operating conditions and introduces the construction of the improved IBRB-a model.

### 2.1. Problem Description

Several issues remain to be addressed when the IBRB is used for fault diagnosis:

Problem 1: Existing data sampling methods have difficulty accurately capturing key fault features under variable operating conditions. This is primarily due to factors: dynamic changes in operating condition parameters induce nonlinear mapping of fault features; environmental factors introduce non-stationary noise, which impairs the reliable identification of key feature regions; and achieving a balance between model efficiency and interpretability is challenging. Therefore, the primary problem for the bearing fault diagnosis system is how to autonomously identify key features stably and efficiently and construct the model reference interval, as follows.

(1) $$Ρ(\hat{H}\in \Omega )<a,\hat{H}=H(x)+\eta$$

where $\hat{H}$ represents the actual extracted fault feature, which is jointly affected by the nonlinear mapping H(x) and non-stationary noise η. Ω is the effective region where the key fault features should lie. a is the preset high-reliability threshold.

Problem 2: When operating conditions change, the rules established on the basis of specific operating conditions fail to accommodate new fault modes and feature mapping relationships, resulting in the formation of “coverage blind areas”. The main reason is that variable operating conditions cause dynamic distortion of the mapping relationship, which prevents adaptation to match new operating conditions and thus leads to a loss of discriminative ability. This dynamic mismatch problem severely restricts the reliability and generalizability of the rule-based system. Therefore, overcoming dynamic changes and ensuring the effectiveness of the diagnostic system under variable operating conditions constitutes a key problem.

(2) $$\begin{array}{l} P_{k}:X\to Y\\ \exists \;x\in X,{\;P\;}_{k}\left(x\right)\;\neq {\;P}_{k^{\prime }}\left(x\right)\;\mathrm{and}\;x\;\notin \;Dom\;\left(P_{k}\right) \end{array}$$

where X is the input feature space and Y is the fault mode output space. k denotes the initial operating condition identifier. $k^{\prime }$ is the new operating condition identifier. $Dom\;\left(P_{k}\right)$ is the effective domain of the initial rule $P_{k}$.

Problem 3: During parameter optimization, it is difficult to achieve convergence to a stable optimal solution, and interpretability is insufficient. Traditional optimization algorithms are designed based on the assumption of fixed extreme points in the objective function. Yet, under variable operating conditions, the objective function’s form changes continuously, leading to drift in the optimal solution and preventing stable convergence. Furthermore, the reasoning process fails to demonstrate the correlation between parameters and diagnostic results, resulting in a lack of interpretability. Consequently, the key problem is to ensure stable convergence to valid solutions and guarantee the transparency of optimization decisions under the condition of a continuously changing objective function.

(3) $$||\hat{\Psi }-\Psi_{*}||>\varepsilon ,\;R\;<\;\tau$$

where Ψ is the optimization parameter, $\Psi_{*}$ is the optimal solution of the objective function that varies under variable operating conditions, $\hat{\Psi }$ is the parameter obtained by the algorithm, R is the correlation degree between the parameters and diagnostic results, ε is the acceptable threshold, and τ is the interpretability threshold.

### 2.2. IBRB-a Methodology Definition

**Figure 1:**
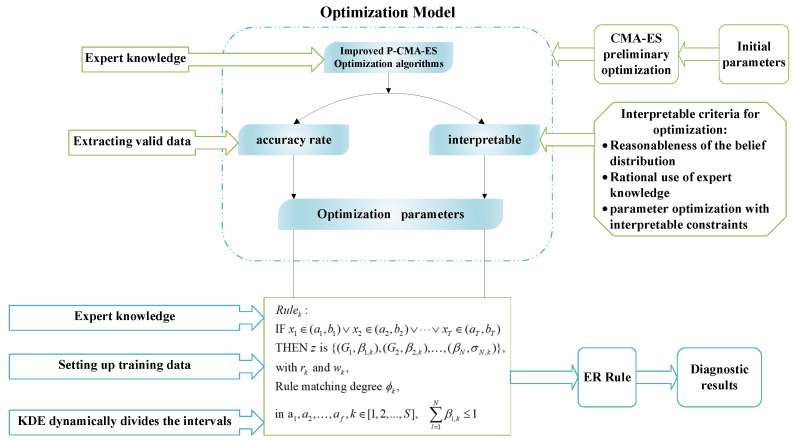
The IBRB-a model’s overall structure.

To address the three aforementioned problems in practical engineering operations, this paper develops a bearing fault diagnosis system founded on IBRB-a. In the novel bearing fault diagnosis system, there are a number of belief rules, and the *k*-th belief rule is formulated as follows:

(4) $$\begin{array}{l} Rule_{k}:\\ \mathrm{IF}\;x_{1}\in (a_{1},b_{1})\vee x_{2}\in (a_{2},b_{2})\vee \ldots \vee x_{T}\in (a_{T},b_{T})\\ \mathrm{THEN}\;z\;\mathrm{is}\;\{(G_{1},\beta_{1,k}),(G_{2},\beta_{2,k}),\ldots ,(\beta_{N},\sigma_{N,k})\},\;\\ \mathrm{with}\;r_{k}\;\mathrm{and}\;w_{k},\\ \mathrm{Rule}\;\mathrm{matching}\;\mathrm{degree}\;\phi_{k},\\ \mathrm{in}\;a_{1},a_{2},\ldots ,a_{f},k\in [1,S],\;\sum_{l=1}^{N}\beta_{i,k}\leq 1 \end{array}$$

where $x_{1},x_{2},\ldots ,x_{T}$ denotes a prerequisite attribute for the bearing failure characteristics to be used as input to the model, T is the number of prerequisite attributes, and S denotes the total number of rules. ${(a}_{i},b_{i})$ is the reference value interval, where i=1,2,…,T, $r_{k}$ denotes the rule reliability. $w_{k}$ represents the rule weights. $a_{1},a_{2},\ldots ,a_{f}$ are interpretable constraints on the model. f indicates the number of interpretable guidelines. $\phi_{k}$ is the rule matching degree factor. The modeling process of the developed bearing fault diagnosis system is shown in Figure 1. It displays the structure of the IBRB-a model and its three primary aspects: modeling, reasoning, and optimization. Therefore, the interpretability should also be balanced from these three aspects.

The description of the interpretability criteria pertaining to the IBRB model in Cao et al. [31]’s paper can be elaborated from three aspects: belief rule base construction, reasoning mechanism, and model optimization, as shown in Table 1. Under variable operating conditions, the bearing fault diagnosis model based on IBRB-a ensures interpretability and reliability in accordance with these criteria. Under such conditions, belief rules can offer explicit semantic descriptions for the input and output of the bearing fault diagnosis, which constitutes the primary expression of the IBRB-a model’s interpretability [32]. By introducing expert knowledge within the model through belief rules, if the optimized belief distribution remains generally consistent with the initial expert settings, the model’s reasoning logic can be considered consistent with domain common sense [33].

**Figure 2:**
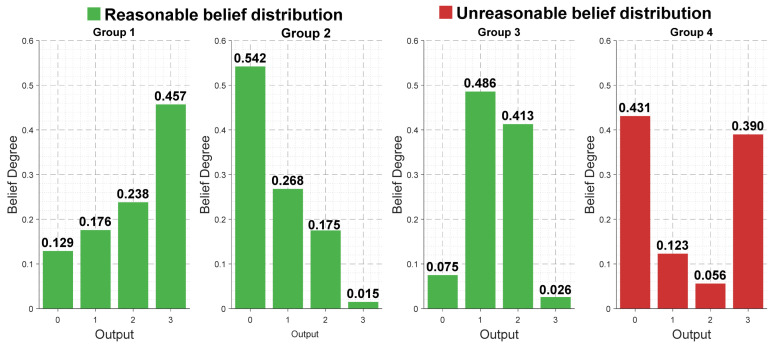
Reasonableness of Belief Distribution.

However, models often improve accuracy at the cost of reduced interpretability, which may lead to unrealistic confidence distributions in the final prediction results. For example, in bearing fault diagnosis, there are four health conditions: normal. A sample output prediction might be: {(Normal, 0.431), (Ball fault, 0.123), (Inner ring fault, 0.056), (Outer ring fault, 0.390)}. This indicates that the system assigns a confidence level of 0.431 to “Normal” and 0.390 to “Outer ring fault”. Such a belief distribution appears contradictory, as a logically consistent outcome should not simultaneously assign high confidence to conflicting conclusions. Therefore, an interpretable belief distribution ought to demonstrate convex or monotonic properties instead of a concave shape, as depicted in Figure 2.

## 3. Reasoning and Optimization of IBRB-a

The IBRB-a model establishes a variable operating condition bearing fault diagnosis framework with the core logic of “feature processing-rule matching-reasoning fusion-parameter optimization”. First, feature selection and KDE-based dynamic division are performed to determine the reference intervals, thereby establishing the foundation for features processing. Then, expert knowledge-based constraints are integrated, the interval belief rule base is initialized, the degree of agreement between samples and intervals is quantified through dynamic rule matching, and initial diagnostic results are produced in combination with the ER strategy. Finally, the improved P-CMA-ES with interpretability constraints is used to optimize the model parameters. Section 3.1, Section 3.2 and Section 3.3 detail the reference interval selection and matching, the model reasoning process, and the parameter optimization method, respectively.

To provide a clear and concise overview of the entire IBRB-a workflow, Algorithms A1 and A2 presents pseudocode for the key steps of feature processing, rule matching, reasoning fusion, and parameter optimization. This algorithmic representation complements the detailed theoretical explanations in Section 3.1, Section 3.2 and Section 3.3.

### 3.1. Dynamic Interval Division

**Figure 3:**
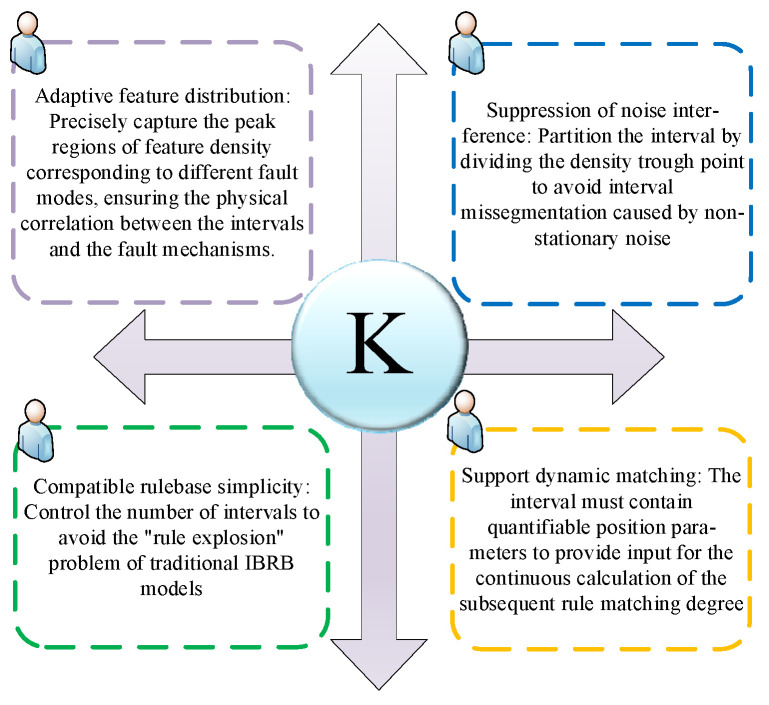
Dynamic interval division multidimensional goals.

Under variable operating conditions, the fault characteristics of bearings exhibit coupled features of nonlinear mapping and non-stationary noise. Traditional fixed interval division methods rely on preset distribution assumptions, which easily lead to under-segmentation of key fault characteristic regions or over-segmentation of redundant regions, making it difficult to balance model efficiency and interpretability. To address this issue, this section combines the multi-dimensional goals for dynamic interval division illustrated in Figure 3 (The symbol *k* shown in the figure is defined as “key”) and proposes a dynamic reference interval division method based on KDE. By analyzing the local density distribution of feature data, the method autonomously identifies interval boundaries, thereby providing a reliable foundation for calculating rule matching degrees.

**Figure 4:**
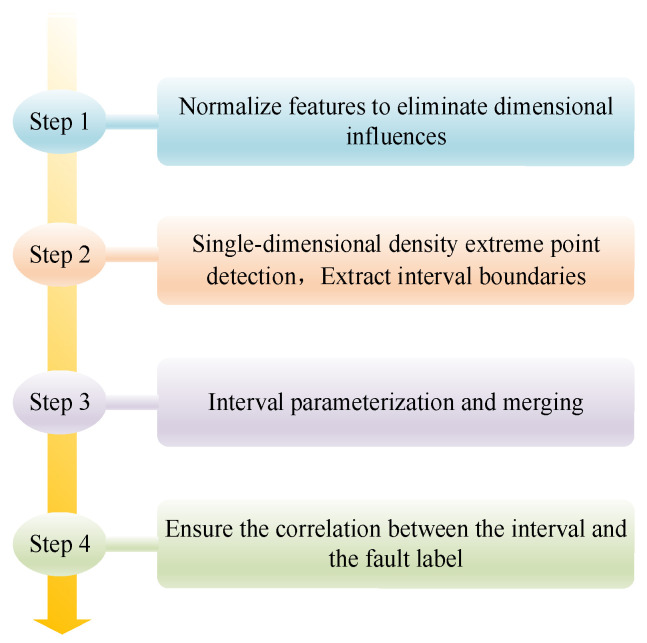
Schematic diagram of the kernel density steps.

On the basis of the filtered n-dimensional fault feature space, KDE is independently performed for each feature dimension, and its mathematical formulation is expressed as:

(5) $${\hat{g}}_{s}(y)=\frac{1}{num\cdot w_{s}}\sum_{k=1}^{num}L\left(\frac{y-y_{k,s}}{w_{s}}\right)$$

where ${\hat{g}}_{s}(y)$ is the probability density estimation function of the *s*-th input feature, which is used to describe the sample distribution density of this feature under different values; num represents the total number of samples; $w_{s}$ is the bandwidth of the *s*-th feature; L(•) is the kernel function; and $y_{k,s}$ is the value of the *k*-th sample on the *s*-th feature, which is directly obtained from the feature extraction results of preprocessed vibration signals of bearings. Combined with the characteristics of bearing fault features, the dynamic interval is partitioned into four key steps as presented in Figure 4.

A Gaussian kernel function is adopted in this study, with its mathematical expression given by:

(6) $$L(u)=\frac{1}{\sqrt{2\pi }}\exp(-\frac{1}{2}u^{2})$$

The Gaussian kernel is characterized by an infinite support set and is infinitely differentiable and smooth, enabling it to generate smooth density estimation curves. Compared to alternative kernels such as the Epanechnikov, triangular, and cosine kernels, the Gaussian kernel provides the optimal balance between capturing fine-grained features of the data distribution and maintaining estimation stability. The bandwidth $W_{s}$ is the most critical parameter in KDE, directly influencing the smoothness of the density estimate. This study employs Silverman’s adaptive rule as the baseline bandwidth:

(7) $$W_{s}=1.06\;\cdot \;\hat{\upsilon }\;\cdot {\;t}^{-1/5}$$

where $\hat{\upsilon }\;$ denotes the sample standard deviation. This rule is considered optimal when the data approximates a normal distribution. Considering that actual bearing fault features may exhibit multimodal distributions, an adaptive adjustment factor ƛ is introduced. The final bandwidth is determined by maximizing the log-likelihood function of leave-one-out cross-validation:

(8) $${W_{s}}^{\prime }=ƛ\;•\;W_{s}\;ƛ\in [0.7,1.5]$$

This strategy ensures adaptive smoothing for different feature distributions, avoiding the extremes of losing feature details or introducing excessive noise. The backend attention mechanism and rule optimization process of the IBRB-a model exhibit a degree of fault tolerance to minor variations in KDE parameters. Even if slight deviations exist in the interval division, the model can compensate through adaptive inference, which structurally guarantees the overall robustness of the method.

This dynamic interval is generated under the drive of KDE, which is consistent with fault mechanisms, adaptive to working condition fluctuations, and free from preset fixed ranges. Based on this, the IBRB-a model obtains adaptive reference intervals, and further establishes a dynamic rule matching degree calculation model to achieve accurate matching between samples and rules.

**Figure 5:**
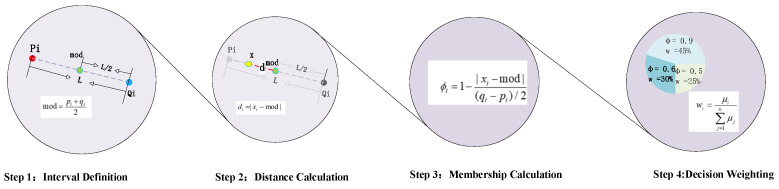
Dynamic rule matching calculation process.

In actual industrial production, the operating conditions of bearings are subject to constant fluctuations. Data such as vibration signal frequency and noise characteristics, which serve as the core basis for bearing fault diagnosis, undergo significant changes, resulting in different manifestations of the same bearing fault under varying operating conditions. However, in the intervals of the traditional IBRB model, when a sample falls within an interval, the interval rules are activated indiscriminately, leading to low sensitivity to faults under different operating conditions. Therefore, this paper proposes a calculation method transitioning from undifferentiated activation to dynamic judgment of activation matching. Let the input sample values of each attribute be denoted as $X=\left\{x_{1},x_{2},\ldots ,x_{T}\right\}$. After interval membership judgment, if the sample falls into the target reference interval, the preset rules within this interval are activated. Meanwhile, the rule matching degree of the sample data is dynamically calculated to quantify the degree of rule activation and the matching level between the sample rules and the interval rules. The corresponding calculation formula is expressed as follows:

(9) $$\begin{array}{l} \phi_{i}=1-\frac{\left|x_{i}-\mathrm{mod}\right|}{(q_{i}-p_{i})/2},\\ x_{i}\in [p_{i},q_{i}],\phi_{i}\in [0,1],\\ where\;\mathrm{mod}=\frac{p_{i}+q_{i}}{2} \end{array}$$

where $\phi_{i}$ represents the matching degree of the sample data of the *i*-th attribute falling into a certain interval matched by the rule, mod represents the center of the reference interval, $\left[p_{i},q_{i}\right]$ represents the interval boundary, and $x_{i}$ represents sample data that falling into this reference interval. This formula takes the midpoint of the interval as the reference benchmark. Through the difference operation and normalization process between the sample coordinates and the midpoint coordinates, it converts the position information of the sample within the interval into a matching degree index in the interval [0, 1]. The calculation result directly reflects the effectiveness of rule activation and the degree of consistency between rules. The central idea of this formula is that the closer the distance between the sample and the center of the reference interval is, the higher the matching degree, which indicates a better degree of agreement between the rule and the current operating condition. When the sample point is infinitely close to the center of the interval, the matching degree approaches the maximum value, and the rule then occupies a dominant position in the decision-making process; conversely, as the distance increases, the matching degree decreases monotonically, and the rule’s influence on the decision-making process gradually diminishes. The calculation process is mainly divided into four steps, as shown in Figure 5.

By calculating the rule matching coefficient within the interval, a method for calculating the weights of activated rules within the interval is designed. When the input sample is located within a specific interval, the method for determining the weights of the activated rules is given by Equation (10):

(10) $$\begin{array}{l} \gamma_{i}=[\gamma_{\min}+\phi_{i}-\gamma_{\min}\times \phi_{i}]\times \gamma \;,\;\\ \left\{\gamma_{\min},\gamma_{i},\gamma \}\in [0,1\right] \end{array}$$

where $\gamma_{i}$ denotes the activation weight of the *i*-th activated interval rule; γ denotes the rule weight; and $\gamma_{\min}$ denotes the minimum activation weight. The primary idea embodied in this formula is that the lower the interval rule matching degree is, the smaller the rule activation weight. $\gamma_{\min}$ is determined on the basis of expert knowledge and can be any constant within the interval [0, 1].

After the above two-step calculation, the interval rule achieves the following effect: When different data sample values fall within the same interval, the rule matching degrees differ, thereby resulting in different performances for different inputs. Therefore, when dividing intervals, the points with the densest distribution should be placed in the middle of the intervals; this allows the highest degree of rule matching to be obtained.

### 3.2. Inference Process of the Model

In complex industrial scenarios, the extensiveness of data and the diversity of expert knowledge are intertwined. To process various types of uncertain information efficiently, the ER inference rules are introduced into the IBRB-a model. The ER rules construct a framework that comprehensively considers the reliability of evidence that effectively describes, transforms, and integrates multisource information in an uncertain environment and ultimately reaches a consistent conclusion. This characteristic enables the IBRB-a model to perform accurate and efficient information processing in complex and dynamic scenarios, provides solid support for the decision-making process, and renders the decision-making basis output by the model more persuasive.

**Figure 6:**
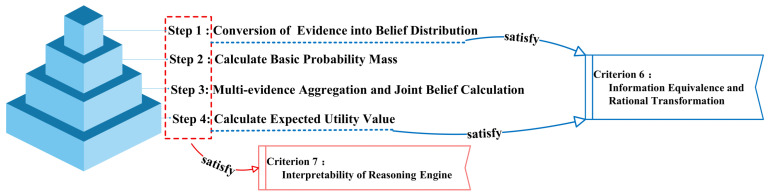
Flowchart of ER rule inference.

While ensuring model reliability and accuracy, the ER rules also prevent the model from falling into a “black-box” or “gray-box” system. During the inference process, as presented in Figure 6, the IBRB-a model achieves a rational transformation of the input and output information (Criterion 6). It not only preserves the informational characteristics of the original samples but also produces inference results that align more closely with real-world scenarios (Criterion 7). This reflects the model’s effective balance between interpretability and performance.

To comprehensively handle the uncertainties arising from multiple rules and diverse information sources, the proposed model employs a two-stage Evidential Reasoning (ER) fusion strategy. The fundamental principle of this strategy is hierarchical information aggregation, which emulates the human expert reasoning process of progressively narrowing down the diagnostic scope to reach a definitive conclusion:

Stage 1: Intra-rule fusion. For a single belief rule, the antecedent attributes may be matched to varying degrees. The objective of this stage is to aggregate these matching degrees across different attributes within the same rule to compute the joint belief degree of the rule being activated as a whole, along with its associated uncertainty.

Stage 2: Inter-rule fusion. After obtaining the joint belief degrees of all activated rules, the objective of this stage is to fuse the potentially conflicting diagnostic conclusions indicated by these different rules, outputting the final belief distribution and the corresponding expected utility value.

**Figure 7:**
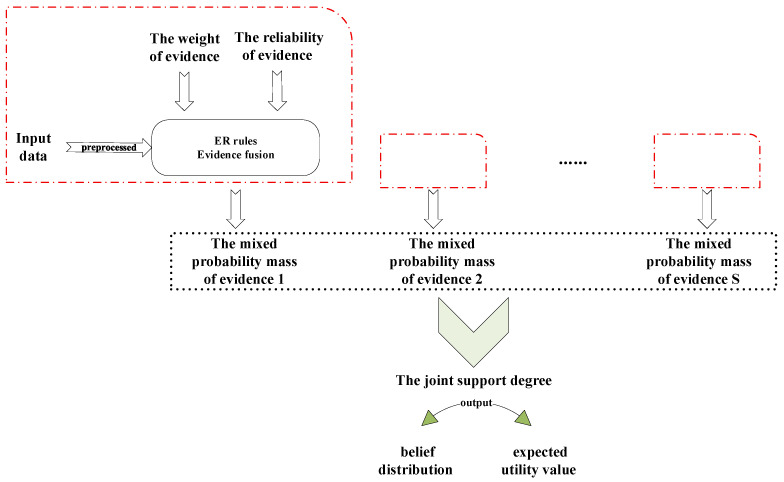
Two-Stage ER Rule Reasoning Flowchart.

This two-stage design ensures the structural clarity of the inference process and the computational tractability. The procedure is visually illustrated in Figure 7 (The content contained in the dashed boxes of the figure is completely identical, and therefore not duplicated for representation). The detailed reasoning steps are elaborated as follows [32].

Step 1: Convert the bearing fault feature data into a belief distribution. The k-th piece of evidence $\delta_{k}(k=1,2,\ldots ,S)$ can be formulated as the subsequent belief distribution.

(11) $$\begin{array}{l} \delta_{k}=\left\{\begin{array}{l} (G_{n},\beta_{n,k}),n=1,2,\ldots ,N;\\ \left(\Theta ,\beta_{\Theta ,k}\right) \end{array}\right\}\\ \mathrm{where}\;0\leq \beta_{n,k}\leq 1\;\mathrm{and}\;\sum_{n=1}^{N}\beta_{n,k}\leq 1\; \end{array}$$

where $\Theta =\{G_{1},G_{2},\ldots ,G_{N}\}$ denotes the frame of discernment; $G_{n}(n=1,2,\ldots ,N)$ denotes the evaluation grades for bearing fault diagnosis results; N denotes the number of evaluation grades; $\beta_{n,k}$ denotes the $G_{n}$ confidence level of the result; and $\beta_{\Theta ,k}$ denotes that the k-th attribute in the frame of discernment is globally unknown.

Step 2: Combine the evidence weight $\omega_{k}(k=1,2,\ldots ,S)$ and reliability $r_{k}(k=1,2,\ldots ,S)$, convert the belief degree into the basic probability mass, and consider normalization, where $\omega_{k}\in [0,1]$ and $r_{k}\in [0,1]$.

(12) $$\begin{array}{l} {\tilde{m}}_{n,k}=\left\{\begin{array}{ll} 0, & G_{n}=\phi \\ b_{rw,k}m_{n,k}, & G_{n}\subseteq \Theta ,\theta_{n}\neq \phi \;\\ b_{rw,k}(1-r_{k}),\; & G_{n}=\beta (\Theta ) \end{array}\right.\\ b_{rw,k}=1/(1+w_{k}-r_{k})\\ m_{n,k}=w_{k}\beta_{n,k} \end{array}$$

where $b_{rw,k}$ is the normalization factor and satisfies condition $\sum_{n=1}^{N}{\tilde{m}}_{n,k}+{\tilde{m}}_{\beta (\Theta ),k}=1$, where $m_{n,k}$ stands for the probability mass of the *k*-th piece of evidence at the specific evaluation grade $G_{n}$.

Step 3: Through iterative aggregation, gradually merge the probability masses of different pieces of evidence, calculate the joint probability, perform normalization, and generate the joint belief degree. The joint support degree $\beta_{G,\delta (k)}$ for S independent pieces of evidence can be calculated as follows:

(13) $$\begin{array}{l} \forall G_{n}\in \Theta ,\\ {\hat{m}}_{n,\delta (k)}=m_{n,\delta (k-1)}-r_{k}\times m_{n,\delta (k-1)}+m_{\beta (\Theta ),\delta (k-1)}m_{n,t}\\ \;\;\;+\sum_{C\cap D=G_{n}}m_{C,\delta (k-1)}m_{D,k}\\ {\hat{m}}_{\beta (\Theta ),\delta (k)}=m_{\beta (\Theta ),\delta (k-1)}-r_{k}\times m_{\beta (\Theta ),\delta (k-1)}\\ m_{n,\delta (k)}=\left\{\begin{array}{ll} 0, & G_{n}=\phi \\ \frac{{\hat{m}}_{n,\delta (k)}}{\sum_{C\subseteq \Theta }{\hat{m}}_{C,\delta (k)}+{\hat{m}}_{\beta (\Theta ),\delta (k)}}, & G_{n}\neq \phi \end{array}\right.\\ \beta_{G,\delta (k)}=\left\{\begin{array}{ll} 0, & G_{n}=\phi \\ \frac{{\hat{m}}_{n,\delta (k)}}{\sum_{C\subseteq \Theta }\mu_{C,\delta (k)}} & G_{n}\neq \phi \end{array}\right.\; \end{array}$$

where k=1,2,…,S; $\beta_{G,\delta (k)}$ denotes the joint support degree of S independent pieces of evidence; and $\delta_{k}$ denotes the joint probability mass of k pieces of evidence and satisfies two conditions: $m_{n,\delta (1)}=m_{n,1}$ and $m_{\beta (\Theta ),\delta (1)}=m_{\beta (\Theta ),1}$. The confidence degree distribution of the relative results conforms to Equation (11).

Step 4: Calculate the weighted sum of the joint belief degree and the utility value in order to get the final diagnosis result. The calculation formula is shown below:

(14) $$Z=\sum_{n=1}^{N}\sigma (G_{n})\beta_{n,\delta (k)}+\sigma (\Theta )\beta_{\Theta ,\delta (k)}$$

where Z is the final utility value, and $\sigma (G_{n})$ is the utility score of $G_{n}$.

### 3.3. Model Optimization

In the current study, the P-CMA-ES optimization algorithm, with its distinctive features, serves as an effective method for optimizing the IBRB prediction model [34]. Its key advantages include: (1) Efficient global search capability, which prevents the algorithm from falling into local optima; (2) simplification of the rule base, which reduces computational costs and avoids the “curse of dimensionality”; and (3) improved reliability through multi-objective balancing that enhances key features. Therefore, the P-CMA-ES is employed in this paper to optimize the IBRB-a model. However, it should be emphasized that increases in model accuracy come at the price of decreased interpretability [35]. To balance accuracy and interpretability, several interpretability constraints [36] are incorporated into the P-CMA-ES framework. The constraints of optimization parameters can be listed as [37].

(15) $$\begin{array}{l} \sum_{n=1}^{N}\beta_{n,k}=1;r_{k}\in [0,1];\\ w_{k}\in [0,1];\beta_{n,k}\in [0,1]\\ where\;n=1,2,\ldots ,N\;\mathrm{and}\;k=1,2,\ldots ,S \end{array}$$

**Figure 8:**
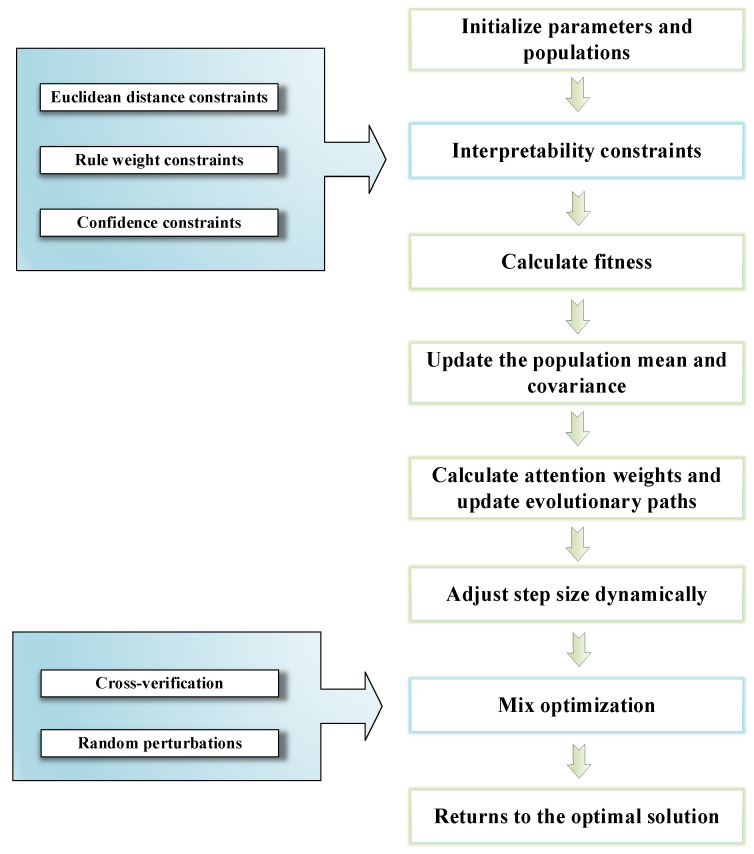
Optimization flowchart.

Within the IBRB-a model, the difference between predicted and actual values is assessed using mean squared error (MSE). The formulation of the objective optimization function is given below [38]:

(16) $$\mathrm{Mse}(\beta ,r,w)=\frac{1}{D}\sum_{k=1}^{D}{\left(U-{U^{*}}_{k}\right)}^{2}$$

where D is the total number of training sample data, U is the model’s output value, and $U^{*}$ is the system’s label value. The optimization process of the improved P-CMA-ES is illustrated in Figure 8. The specific operation steps are as follows:

Step 1: Parameter input. In the IBRB-a model, the parameters to be optimized include confidence, rule reliability, and rule weight. The parameter set can be expressed as

(17) $$\Psi^{0}=\lambda^{0}(\beta ,\omega ,r)\;$$

where $\Psi^{0}$ represents the initial parameter set.

Step 2: To enhance the diversity of the initial population and prevent the algorithm from falling into local optima, Gaussian random perturbations are introduced based on the initial point $x_{k}^{(g+1)\;}$ to generate u individuals of the initial population:

(18) $$x_{k}^{(g+1)\;}=m^{\left(g\right)}+\vartheta z_{k},k=1,2,\ldots ,u$$

where $x_{k}^{(g+1)\;}$ is the k-th individual in the g+1 generation, ϑ is the disturbance coefficient, which governs the amplitude of the disturbance, $z_{k}\sim N(0,I)$ is a random vector following the standard normal distribution, u denotes the number of additional individuals in the initial population. The original initial points and perturbed points are merged to calculate the mean value of the initial population.

(19) $${\bar{x}}^{\left(g\right)}=\frac{1}{v+1}(x^{\left(g\right)}+\sum_{k=1}^{u}x_{k}^{(g+1)\;})$$

This mean value is employed as the initial search center of the P-CMA-ES algorithm.

Step 3: Add interpretability constraints.

(1) Euclidean distance constraint: Expert knowledge is among the core elements of interpretability, and the optimization process of local search needs to be anchored in expert judgment. To this end, expert knowledge can be integrated into the initial population, and further local optimization can be carried out with the help of the Euclidean distance [39]:

(20) $$\xi^{\left(g\right)}=\left\{\begin{array}{l} EXP,ifs=1\\ \xi^{\left(g\right)},ifs\neq 1 \end{array}\right.$$

where $\xi^{\left(g\right)}$ denotes the population of the g generation and EXP represents expert knowledge. To ensure that the optimized parameters maintain a reasonable distance from the initial values, a Euclidean distance constraint is imposed on the parameters, and its expression is as follows [32]:

(21) $$\sqrt{\sum_{i=1}^{n}{\left(eq_{mean,i}-eq_{k,i}\right)}^{2}}\leq d$$

where ${eq}_{mean}$ is the mean value of the current population; $eq_{k}$ is the newly generated individual; and d is the maximum allowable distance set by experts. Through the distance threshold d set by experts, the search range of the optimization algorithm can be effectively controlled, the risk of parameter solutions deviating from expert cognition can be reduced, and thus, the random oscillation phenomenon in the optimization process can be suppressed.

(2) Rule parameter constraint: For the inactive rule k∈P in the rule set, its parameter vector must satisfy:

(22) $$can_{k}(I_{k})\;=\;can_{0}(I_{k})\;;\;\forall \;k\in K$$

where $can_{k}$ denotes the *k*-th candidate solution vector; $can_{0}$ represents the initial population vector; K represents the index set of inactive rules; and $I_{k}$ denotes the index range of the parameters corresponding to the *k*-th rule in the vector.

(3) Attribute weight constraint: To ensure that the attribute weights are not lower than the minimum values set by experts, the following constraint is imposed.

(23) $$\omega_{k}\geq \omega_{k,\min}$$

where $\omega_{k}$ denotes the weight of the *k*-th attribute and where $\omega_{k,\min}$ is the minimum threshold of the attribute weights.

(4) Confidence constraint: The confidence constraint is reflected in two main aspects. First, it is the constraint on the variation range of confidence: the variation range of the optimized confidence should not exceed the threshold, which satisfies the following formula [36].

(24) $$|\beta_{k}\;-\beta_{0}\;\;|\leq v$$

where $\beta_{k}$ denotes the confidence value during the optimization process; $\beta_{0}$ represents the initial confidence value; and v denotes the maximum threshold for confidence variation. Second, the normal-like distribution shape constraint: To ensure that the confidence variation conforms to a reasonable probability distribution pattern, the confidence vector $\beta =[\beta_{1}\;,\beta_{2}\;,\beta_{3}\;,\beta_{4}\;]$ of each evaluation grade must satisfy one of the following four monotonic patterns [40].

Monotonic increase: $\beta_{1}\;\leq \beta_{2}\;\leq \beta_{3\;}\leq \beta_{4}$.

First increase then decrease: $\beta_{1}\;\leq \beta_{2}\;\geq \beta_{3\;}\leq \beta_{4}$.

Unimodal distribution: $\beta_{1}\;\leq \beta_{2}\;\leq \beta_{3\;}\geq \beta_{4}$.

Monotonic decrease: $\beta_{1}\;\geq \beta_{2}\;\geq \beta_{3\;}\geq \beta_{4}$.

This constraint ensures that the confidence distribution conforms to the normal-like characteristics common in decision logic. If the optimization result does not satisfy the above patterns, the confidence should be adjusted to meet the constraint.

Step 4: Projection Constraint. The projection operator is used to ensure that the solution vector satisfies the following formula:

(25) $$A_{eq}\vec{x}=b_{eq},\vec{x}\geq 0$$

where $A_{eq}$ is the equality constraint matrix; $b_{eq}$ is the right-hand side vector of the equality constraint; and $\vec{x}$ is the solution vector, which includes confidence, rule weights, and attribute weights. The projection algorithm’s specific steps are outlined below:

(1) Orthogonal projection onto a hyperplane: For each set of constraints $S_{i}$, calculate the vertical distance between the current solution and the canonical hyperplane $\sum x_{j}=1$ and project it onto the constraint plane along the normal vector direction, as in Equation (26):

(26) $$\begin{array}{l} {\vec{x}}_{S_{i}}\leftarrow {\vec{x}}_{S_{i}}-\frac{1}{\left|S_{i}\right|}(\sum_{j\in S_{i}}{\vec{x}}_{j}-b_{i})A_{i}\\ A_{i}={\left[1,1,\ldots ,1\right]}^{T}\in {\mathbb{R}}^{\left|S_{i}\right|} \end{array}$$

where $b_{i}$ is the target value of the i-th constraint.

(2) Nonpositive repair: Set the nonpositive components to zero and uniformly compensate for the total sum of the nonpositive values to the positive components. This operation ensures $x_{j}\geq 0$ while maintaining the constraint conditions of $\sum_{j\in S_{i}}{\vec{x}}_{j}=1$, and its expression is as follows:

(27) $$\begin{array}{l} {\vec{x}}_{j}=\max(0,{\vec{x}}_{j}),\forall j\in S_{i}\\ \partial =\sum_{j\in S_{i}}\max(0,-{\vec{x}}_{j}^{old})\\ {\vec{x}}_{j}\leftarrow {\vec{x}}_{j}+\frac{\delta }{n_{j}} \end{array}$$

where $n_{j}$ is the number of nonzero components in $S_{i}$ and where ∂ is the sum of deviations caused by the correction of negative components.

(3) Loop processing: Repeat the above process for each confidence distribution constraint until all the constraints satisfy the linear constraints and nonnegativity constraints.

Step 5: Optimal Solution Selection and Mean Update. Among the offspring generated in each generation, the top b optimal individuals are selected through fitness ranking for weighted recombination, and the population mean is updated as follows:

(28) $$\begin{array}{l} \xi_{mean}^{\left(g+1\right)}=\sum_{i=1}^{\mu }\varpi_{i}\xi_{i:\lambda }^{\left(g\right)}\;,\\ \varpi_{i}=\frac{\log(\mu +0.5)-\log i}{\sum_{j=1}^{\mu }\left(\log(\mu +0.5)-\log j\right)}\\ \lambda =10+\lfloor 3\log n\rfloor \;,\\ \;\mu =\lfloor \lambda /2\rfloor \end{array}$$

where $\xi_{i:\lambda }^{\left(g\right)}$ is the *i*-th optimal individual in the fitness ranking of the *g*-th generation. $\varpi_{i}$ is the exponential decay weight; μ is the number of preferred individuals; λ is the number of offspring. In addition, attention weights are introduced to enhance the contribution of important individuals. The attention formula is as follow:

(29) $$x_{\mathrm{mean}}^{\mathrm{attn}}=\sum_{i=1}^{\mu }\alpha_{i}x_{i:\lambda }^{\left(g\right)}\;,\;\alpha_{i}=\frac{\exp(-f(x_{i}))}{\sum_{j=1}^{\mu }\exp(-f(x_{j}))}\;$$

where f(⋅) is the fitness function; the negative exponential transformation ensures that individuals with higher fitness values obtain greater weights.

Step 6: The P-CMA-ES guides the search direction by adaptively updating the covariance matrix C, and its update mechanism combines evolutionary path accumulation, multiparent information integration, and constraint handling techniques. The update formula as in Equation (30):

(30) $$\begin{array}{ll} C^{\left(g+1\right)}= & (1-c_{1}-c_{\mu })C^{\left(g\right)}+\\ & c_{1}\left[path_{c}^{\left(g+1\right)}{\left(path_{c}^{\left(g+1\right)}\right)}^{⊤}+(1-h_{\sigma })\cdot c_{c}(2-c_{c})C^{\left(g\right)}\right]+\\ & c_{\mu }\sum_{i=1}^{\mu }\alpha_{i}\times Z_{i}{\left(Z_{i}\right)}^{T} \end{array}$$

where C is the covariance matrix, which determines the shape and direction of the search distribution; $c_{1}$ is the rank-one update learning rate; $c_{\mu }$ is the rank −μ update learning rate; $path_{c}$ is the evolutionary path of the covariance matrix; $h_{\sigma }$ is the heuristic indicator, which takes a value of 0 or 1; $Z_{i}$ is the accumulation coefficient of the evolutionary path; $\alpha_{i}$ is the attention weight of the *i*-th parent; $c_{c}$$Z_{i}$ is the standardized mutation vector [41], and $Z_{i}$ satisfies Equation (31).

(31) $$Z_{i}=\frac{{\vec{x}}_{i:\lambda }^{\left(g\right)}}{step^{\left(g\right)}}-\frac{m^{\left(g\right)}}{step^{\left(g\right)}}$$

where ${\vec{x}}_{i:\lambda }^{\left(g\right)}$ is the “candidate solution” explored by the algorithm in the search space; $m^{\left(g\right)}$ is the mean vector of the population in the *g*-th generation; and $step^{\left(g\right)}$ is the step size of the *g*-th generation.

## 4. Experimental Validation

In this section, experiments are performed under variable load conditions using Case Western Reserve University (CWRU) and Southeast University (SEU) datasets, aiming to evaluate the accuracy, interpretability, and generalization of the proposed IBRB-a fault diagnosis model.

### 4.1. Evaluation Indicators

Under variable operating conditions of bearings, various parameters and external factors are subject to random variations. Accuracy is associated with the safety of equipment operation and production efficiency; interpretability enables technicians to verify and adopt the model’s diagnostic recommendations; and strong generalization capability enables the transfer of learned knowledge to new operating conditions and unseen fault types—this ultimately determines the model’s practical utility in real industrial environments.

To validate the model’s effectiveness in bearing fault diagnosis, we select four evaluation metrics for testing. The first metric is the overall accuracy (ACC) [42], can be described as follows:

(32) $$ACC=\frac{\sum_{i=1}^{N}TP_{i}}{\sum_{i=1}^{N}\left(TP_{i}+FP_{i}+FN_{i}+TN_{i}\right)}$$

where $TP_{i}$ is the number of true positives for category $G_{i}$; $FP_{i}$ denotes the count of true positives for category $G_{i}$; $FN_{i}$ is the number of false positives for category $G_{i}$; and $TN_{i}$ denotes the count of true negatives for category $G_{i}$. The second evaluation indicator is the positive predictive value (PP) [43]:

(33) $$PP_{i}=\frac{TP_{i}}{TP_{i}+FP_{i}}\;$$

is calculated separately for $G_{i}$ categories. where the PP_a_ for all categories are averaged to obtain the following:

(34) $$PP_{a}=\frac{1}{N}\sum_{i=1}^{N}PP$$

The third indicator is the true positive rate (Recall) [44,45]:

(35) Recalli=TPiTPi+FNi

is calculated separately for $G_{i}$ categories. where the recalls for all categories are averaged to obtain the values:

(36) Recalla=1N∑i=1NRecalli

The fourth metric is the F1 score, which stands for the harmonic mean of accuracy and recall, and it is calculated via the following formula:

(37) F1i=2×(PPi×Recalli)PPi+Recalli

is calculated separately for $G_{i}$ categories. where the F1_a_ for all categories are averaged to obtain the values:

(38) $${F1}_{a}=\frac{1}{N}\sum_{i=1}^{N}F1_{i}$$

The F1 score spans from 0 to 1, with a value closer to 1 signifying superior fault diagnosis performance in terms of balancing precision and recall.

### 4.2. Data Introduction

#### 4.2.1. CWRU Datasets

The dataset used for experimental validation incorporated four conditions: normal, ball faults, inner ring faults, and outer ring faults. The condition labels presented in Table 2. Vibration signals were collected with a sampling frequency of 12 kHz under different motor load conditions—0, 1, 2, and 3H—to capture behavioral characteristics under varying operational states.

In this experiment, a bearing with a radius of 0.007 mm was used as an example, and the data were divided into four groups for verification under the same rotational speed (1200 r·min^−1^) while different loads [46]. Detailed grouping information is presented in Table 3. Under 0H load, the first 121,265 data points were extracted from each fault type’s vibration signals and then separated into 118 sample groups. Each experiment included 472 samples (118 data points × 4 fault types). Of all samples, 70% were selected at random for model training, and the remaining 30% served for testing. Similarly, data extracted from the other three load groups also correspond to a four-class classification task.

#### 4.2.2. SEU Bearing Data

In addition to the CWRU bearing dataset, SEU were also used for verification. This dataset was collected under the operating conditions of a rotational speed system load set to 20 Hz–0 V, encompassing 3 types of single-bearing fault states, 1 type of mixed fault state, and 1 type of healthy state. For the purpose of this paper, only data corresponding to 3 types of single faults and the healthy state were selected to verify the reliability and generalizability of the IBRB-a model. The grouping information is detailed in Table 4, and the label values of the fault states are presented in Table 2.

### 4.3. Data Preprocessing

Both datasets consist of bearing vibration signals actually collected from industrial test benches and are susceptible to interference from sensor noise, environmental vibration noise, and other sources. Such interference may obscure the fault characteristics of bearings, compromise the accuracy of feature extraction, and degrade the performance of subsequent diagnostic models. Thus, data preprocessing involving denoising and dimensionality reduction serves as a critical prerequisite for improving data quality and ensuring the reliability of diagnosis.

**Figure 9:**
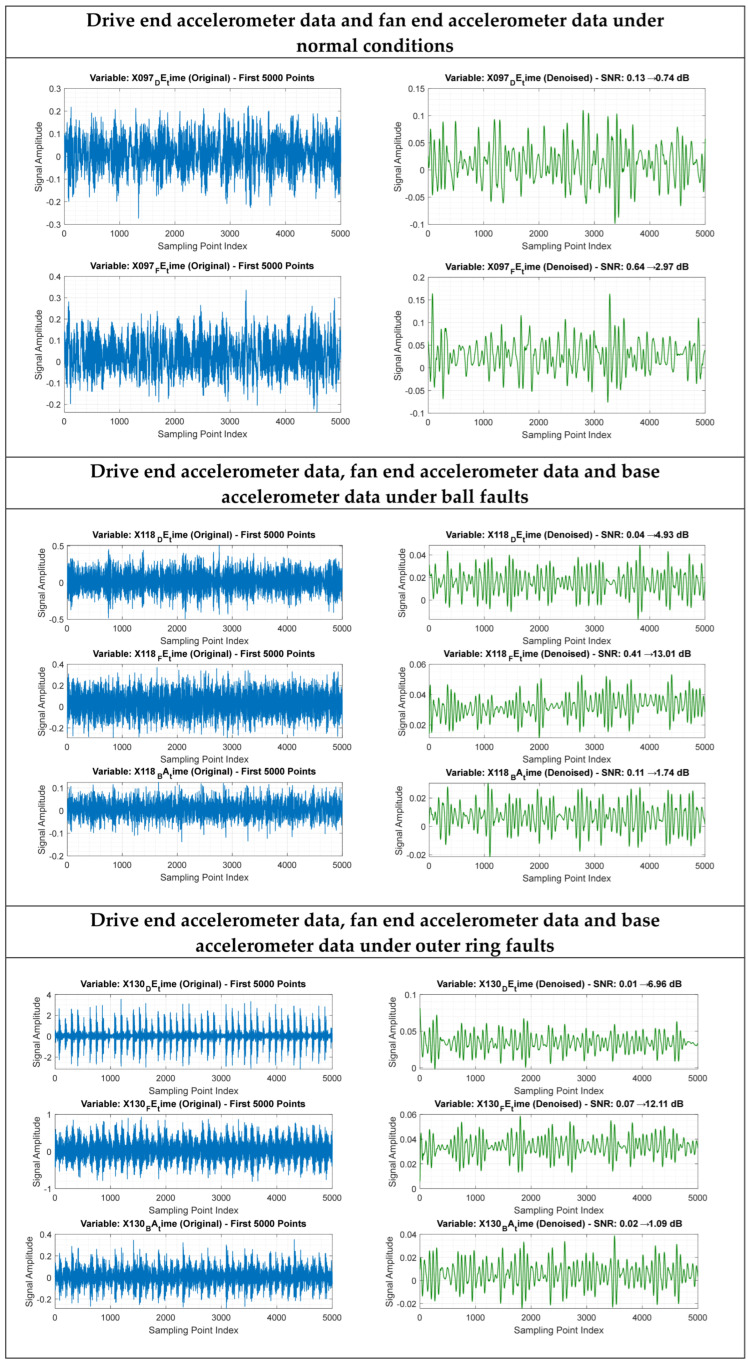

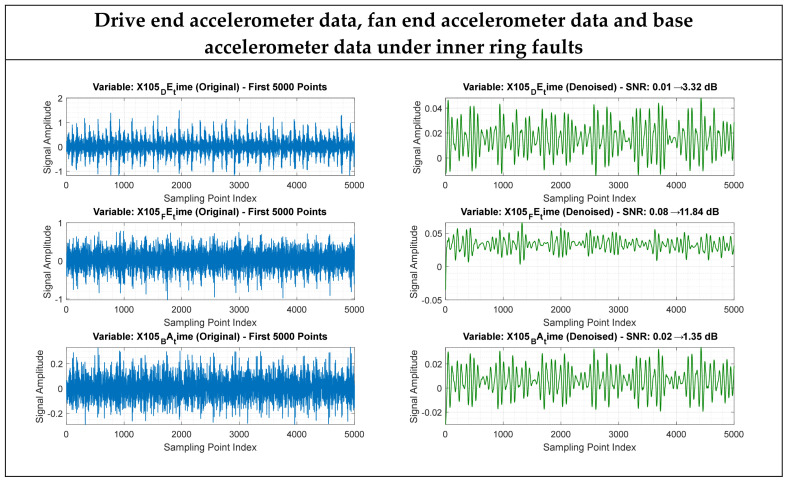
Before and After Noise Reduction.

Wavelet threshold denoising is employed in the experiment to eliminate high-frequency noise from raw vibration signals, which exhibits superior performance in processing non-stationary signals such as bearing vibrations. Specifically, 5-level decomposition based on the db4 wavelet basis is adopted, combined with soft thresholding denoising using the square root logarithm (sqtwolog) adaptive threshold; after signal reconstruction, the length is calibrated to ensure consistency. Subsequently, time-frequency domain features are extracted, and dimensionality reduction is implemented via Principal Component Analysis (PCA) to simplify the model and enhance its generalization ability. As shown in Figure 9, the signal-to-noise ratio (SNR) of the denoised signal is significantly improved with clearer fault characteristics; after dimensionality reduction, the feature dimension is greatly reduced while the discriminative ability is retained. This preprocessing approach effectively improves data quality and lays a solid foundation for accurate fault diagnosis.

**Figure 10:**
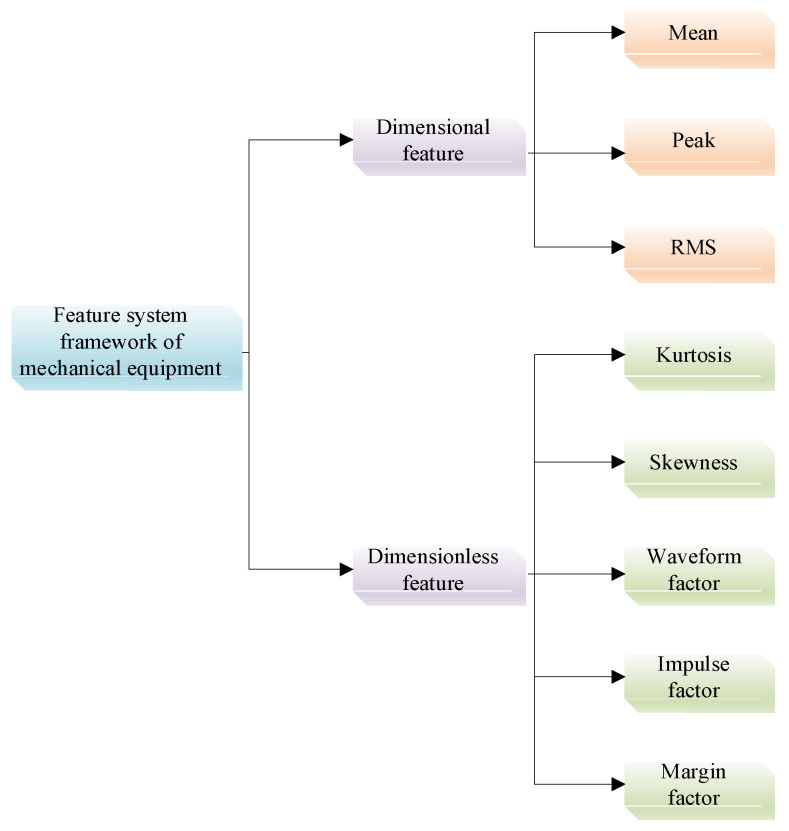
Equipment Vibration Signal Characteristic System Framework.

After noise reduction, a time-domain feature extraction method similar to that proposed in Reference [47] was adopted. After performing time-domain analysis on the bearing vibration signals, the resulting feature framework is illustrated in Figure 10. In-depth examination of these time-domain feature parameters enables accurate identification of fault types under different operating conditions, thereby contributing to the diagnostic method’s reliability and accuracy.

**Figure 11:**
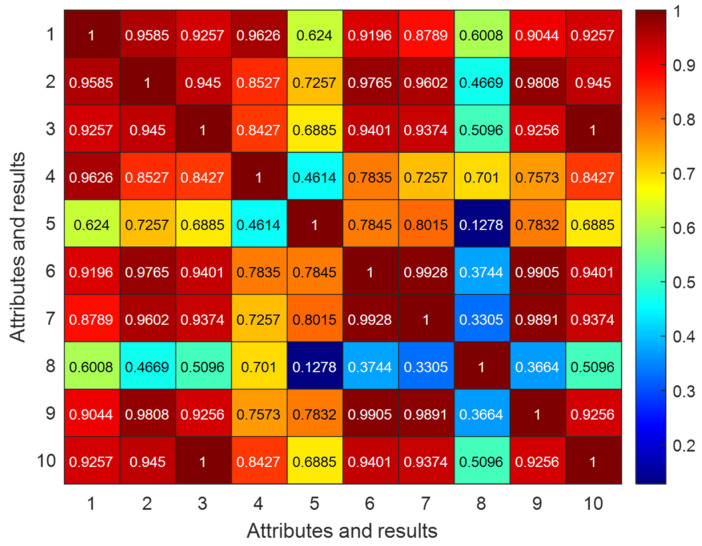
Correlation between Features and Labels Under 0H.

To reduce the interference of high-dimensional feature redundancy on model performance while retaining key information strongly correlated with fault modes, it is necessary to screen the original feature set. This set consists of eight time-domain feature parameters, as shown in Figure 10. The Pearson correlation coefficient was employed to analyze the correlation among all features. The visualization results under the 0H condition are presented in Figure 11, and the correlation coefficients between each feature and the fault label are detailed in Table 5. The four features with the highest absolute correlation coefficient values—peak, RMS, waveform factor and impulse factor—were selected as input variables. This approach reduces the input dimensionality of the model while ensuring the effective retention of core fault-related information.

### 4.4. CWRU Dataset Validation

#### 4.4.1. Model Prediction and Validation

This section elaborates only on bearing fault diagnosis under the 0-load condition in detail, and the processing procedures for other loads are analogous. The *k*-th rule of the bearing fault diagnosis model based on IBRB-a as in Equation (39).

(39) Rulek:IFPeak∈(a1,b1)∨RMS∈(a2,b2)∨WaveformFactor∈(a3,b3)∨ImpulseFactor∈(a4,b4),Then power fault detection results are(G1,β1,k),(G2,β2,k),(G3,β3,k),(G4,β4,k), with rk and ωk ,rule matching degree ϕk,in interpretable constraint a1,a2,…,af,k=(1,2,…,S),∑n=14βn,k≤1

where $G_{1},G_{2},G_{3},G_{4}$ represents four evaluation grades.

The model’s initial parameters are presented in Table 6. Among these, the constrains for rule reliability and rule weight were determined by domain experts on the basis of prior knowledge. First, the model parameters were initially optimized via the standard covariance matrix adaptation evolution strategy (CMA-ES) algorithm, and the optimized parameter configuration is shown in Table 7. The initially optimized parameters were subsequently input into the improved P-CMA-ES for further optimization. The final parameter values of the model are shown in Table 8. The initial parameter setting strategy of this optimization algorithm and the corresponding parameter values are provided in Table 9.

**Figure 12:**
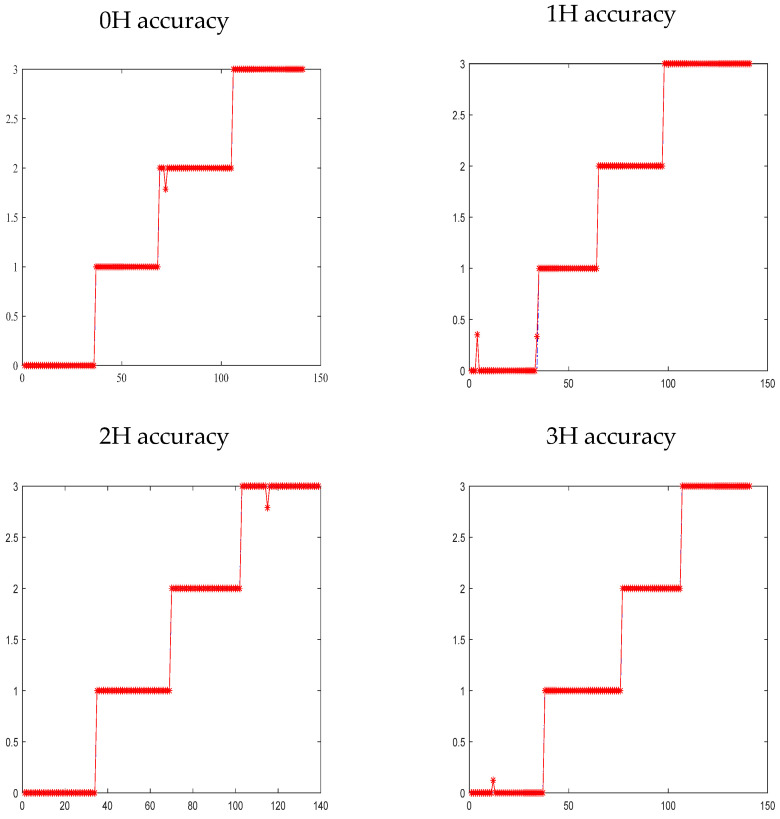
Accuracy Under Each Load.

**Figure 13:**
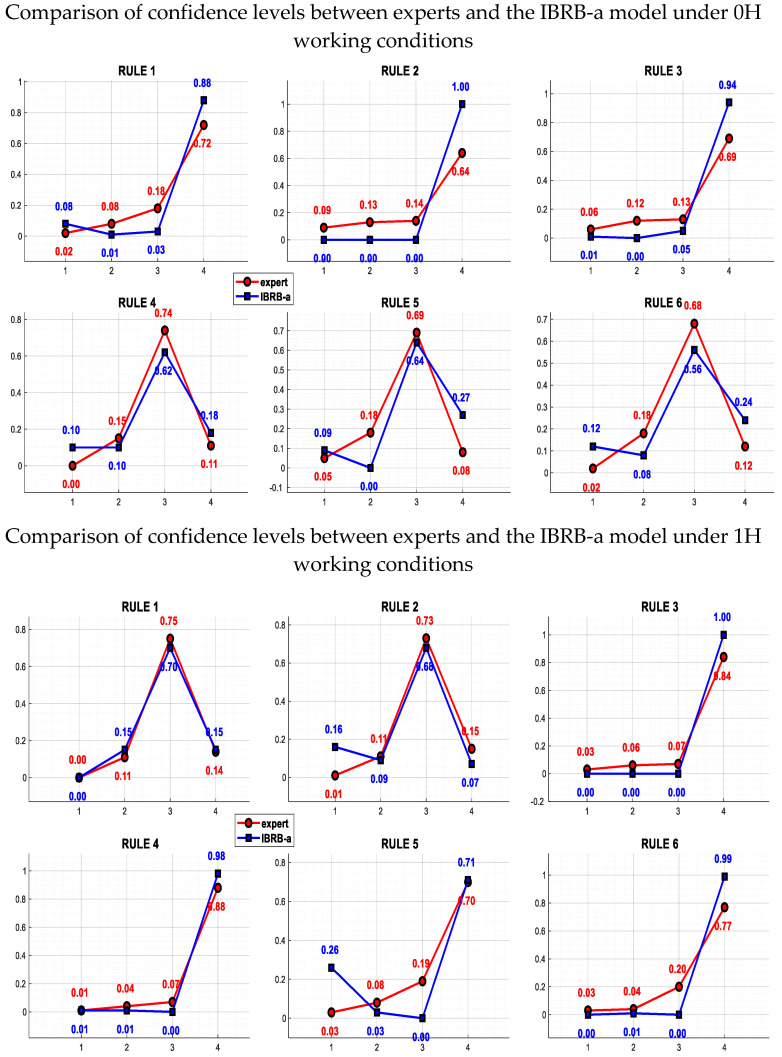

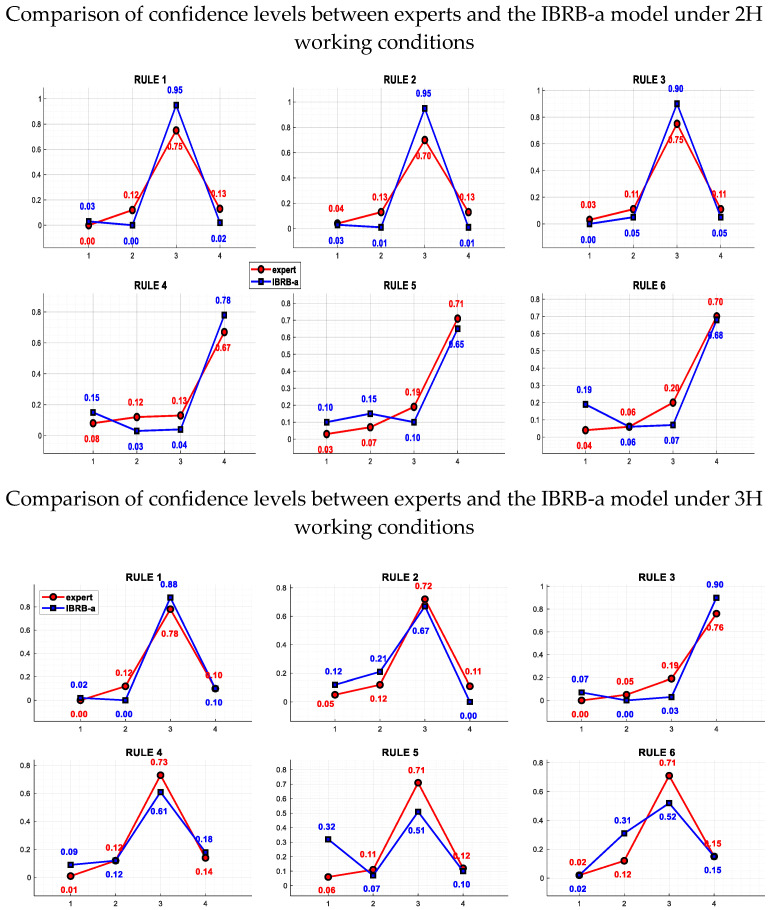
Confidence distribution of the output under each load.

(1) Parameter setting: The constrained P-CMA-ES was employed to optimize the model. The optimization ranges of the rule weights and rule reliability were constrained to the interval [0.5, 1]. Moreover, the belief degrees corresponding to the consequence part of each rule were also constrained to vary within a specified range around their initial values. The optimized parameters are summarized in Table 6. All optimized values remain within the bounds defined by expert knowledge, ensuring that the resulting parameters of the IBRB-a model are both reasonable and suitable for practical application.

(2) Curve Fitting Results of the Model’s Predicted Diagnostic Outcomes and Actual Values: Under the four operating conditions, the fitting results between the model’s predicted values and actual values are shown in Figure 12, where the horizontal axis represents the sample number and the vertical axis denotes the classification number. This effect is attributed to the dynamic rule matching degree and the novel rule weight calculation method adopted by the model—these two elements endow the model with stronger adaptability, enabling it to smoothly cope with data fluctuations and nonlinear trends. As shown in the first subplot of Figure 12, the predicted values of the IBRB-a model exhibit an extremely high degree of fit with the actual labels. The accuracy reaches 99.29% under the 0-load condition. Table 8 clearly presents the IBRB-a fault diagnosis results under the 0H operating condition, which demonstrates high fault identification accuracy.

(3) Model interpretability: As shown in the first subplot of Figure 13, under the effect of the P-CMA-ES with expert constraints, the IBRB-a model retains parameter interpretability. This enables the belief distribution of its output results to be highly consistent with the initial expert-defined belief distribution. The study also reveals that even when complex algorithmic optimization is employed to increase model accuracy, the IBRB-a model continues to accurately reflect expert knowledge and judgments. This not only ensures the interpretability of the diagnostic results but also guarantees the transparency of the process and the reliability of the outcomes.

#### 4.4.2. Results

The results of experimental show that the predicted values of the IBRB-a model show strong consistency with the actual labels in terms of fitting performance. The prediction accuracy is summarized in Figure 12. Under the four distinct load conditions, the model’s accuracy rates of 99.29%, 98.67%, 99.29%, and 99.29%. The overall variation in accuracy remains within a narrow range of 0.62%, indicating high stability under dynamic load changes. These results outperform those reported in comparable studies under similar variable operating conditions.

Analyze the IBRB-a model’s fault diagnosis results across the 0H, 1H, 2H, and 3H operating conditions. Table 10, Table 11, Table 12 and Table 13 show that the model maintains high fault identification accuracy across all operating conditions. Among these results, the positive predictive values (PPs) for most fault categories (e.g., G1, G2, and G4) under different operating conditions are close to or equal to 99%, while recall values remain consistently above 94%. These results systematically demonstrate the model’s ability to stably identify fault modes under different load conditions, providing a robust experimental foundation for its application in complex operational environments.

Further analysis of the dynamic behavior of performance indicators reveals that the influence of operational disturbances on diagnostic performance varies significantly across fault categories. Specifically, the G2 fault category demonstrates greater sensitivity to changes in operating conditions: its recall is 0.97 under the 0H condition, declines slightly to 0.94 under 1H operating condition, and recovers to 1.0 under 2H condition. Moreover, the positive predictive value (PP) of G2 decreases briefly to 0.94 under 1H operating condition but remains stable at 1.0 under all other conditions. These fluctuations reflect the complex coupling relationship between fault characteristics and operating parameters. The data indicate that the G2 fault mode is more susceptible to load variations. It is inferred that this behavior may be linked to nonlinear distortions in the vibration spectrum characteristics of this fault type within certain load ranges. These observations provide a basis for subsequent model refinement targeting specific fault modes.

Regarding interpretability, the IBRB-a model is underpinned by an optimization algorithm constrained by expert knowledge and an attention mechanism based on structured embedding. First, as elaborated in Section 3.3, the expert-constrained P-CMA-ES algorithm ensures that all rule parameters remain within intervals with clear physical meanings post-optimization, thereby preserving the semantic interpretability of the parameters at the source. Furthermore, as illustrated in Figure 13, the “feature-confidence” distribution generated by the model during inference is not a black-box output, and its decision-making logic is fully traceable: the distribution of attention weights directly reveals which features play a critical role in activating specific rules for a given input, while the confidence distribution of the rules yields the final diagnostic conclusion. This transparent inference chain—from input features to attention weights, followed by rule activation and conclusion fusion—endows each diagnostic decision with explicit mechanistic analyzability, mitigating the “black-box” effect inherent in complex models and establishing a solid foundation for the credible deployment and iterative optimization of the model in practical engineering applications.

**Figure 14:**
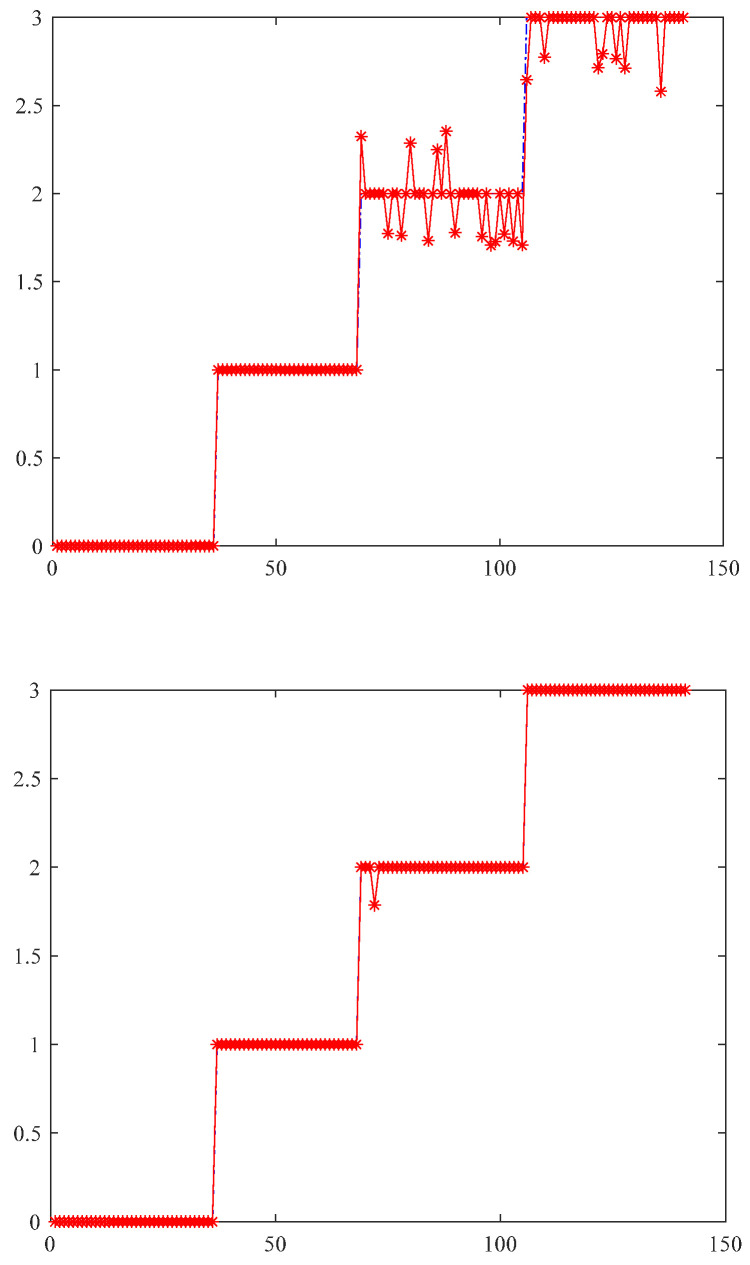
Accuracy comparison with or without random disturbances under 0H conditions.

Furthermore, the initialization random perturbation strategy proposed in this study not only enhances population diversity but also improves algorithm robustness. On the premise of preserving the core characteristics of expert knowledge, the optimization success rate is significantly improved by introducing random perturbations. Taking the 0H operating condition as an example, the accuracy rate is increased from 85.11% to 99.29%, as illustrated in Figure 14, where the horizontal axis represents the sample number and the vertical axis denotes the classification number. This reflects an effective balance between “respecting expert knowledge” and “enhancing algorithm robustness” in interpretable optimization. The results indicate that the method proposed in this study can adapt to the input of different initial expert knowledge in practical applications, thus providing stable and reliable technical support for the optimization of expert knowledge-based intelligent systems.

### 4.5. SEU Dataset Validation

To further test the generalization ability and reliability of the proposed method, a validation experiment was conducted using a distinct dataset. This dataset exhibits substantial differences in sample distribution compared to the first dataset and includes 473 fault samples covering four fault categories (G1–G4) under load operating conditions of 20 Hz–0 V. The experimental processing workflow strictly followed the standard paradigm of the first set of experiments: initial parameters were set on the basis of expert knowledge, followed by preliminary optimization via the conventional CMA-ES algorithm and subsequent iterative optimization via the P-CMA-ES. The experimental results are presented below.

(1) Parameter Optimization and Constraint Satisfaction: The initial parameters for the new dataset were set on the basis of experts’ prior knowledge of its fault characteristics. The constraint settings remained consistent with the first experiment: the optimization ranges for both rule weights and rule reliability were constrained to [0.5, 1], and the belief degrees were limited to vary within their initial value intervals. The parameter values after preliminary optimization via the conventional CMA-ES are provided in Table 14, while the final parameters optimized via the P-CMA-ES are detailed in Table 15.

The analysis indicates that all optimized parameters for the new dataset strictly fall within the expert-defined constraint boundaries. This result confirms that even under varying data distributions and fault characteristics, the P-CMA-ES maintains stable compliance with expert knowledge constraints, with no constraint violations observed. This finding verifies the method’s reliability during the parameter optimization process.

**Figure 15:**
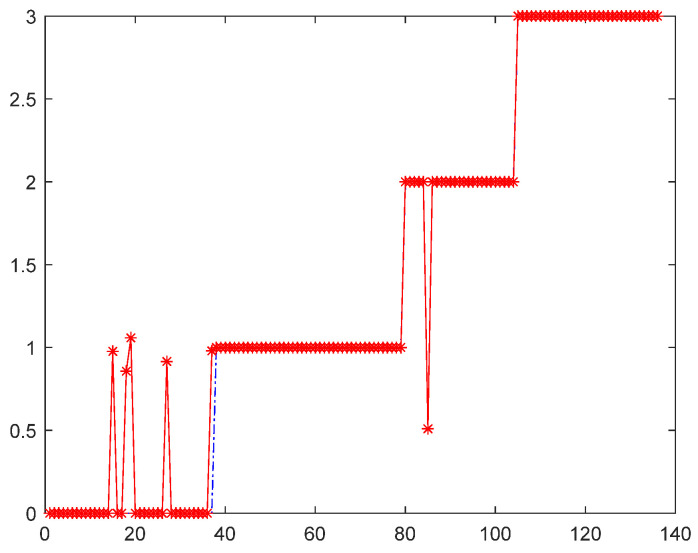
Accuracy at 20 Hz–0 V.

**Figure 16:**
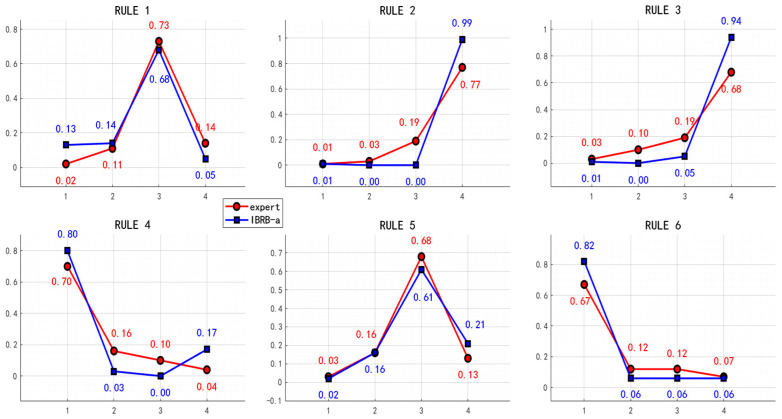
Confidence distribution of the 0 V output.

(2) Model diagnostic performance: On the new dataset, the agreement between the predicted values and true values of the IBRB-a model is presented in Figure 15. The results indicate that the model maintains high fitting accuracy on this dataset, accuracy reaches 95.59%. This indicates that the method retains stable performance even under completely different data distributions. As shown in Table 16, the fault identification accuracy under these operating conditions further verifies the applicability of the proposed method.

(3) To verify whether the model preserves knowledge interpretability on new datasets, we compared the optimized confidence distributions with the initial expert settings. As depicted in Figure 16, the optimized distributions exhibit a high degree of consistency with expert specifications regarding the confidence ranking of critical fault modes and their associated dominant features. For instance, rules initialized with high confidence by experts for “outer race faults” retained top rankings after optimization on new data, with no semantic deviation observed in their associated features. This indicates that the optimization process adapts to new data distributions while preserving the core framework and semantic structure of expert knowledge, without sacrificing interpretability for performance gains. Consequently, the model’s interpretability trait remains robust when generalized to new operating conditions.

(4) Robustness Analysis: The optimization algorithm maintains stable performance on new datasets, exhibiting strong cross-dataset generalization ability and anti-interference robustness, thus providing a reliability guarantee for its deployment in practical applications.

Verification results on the second dataset indicate that the proposed method maintains stable diagnostic accuracy, adherence to parameter constraints, and reliable parameter interpretability and robustness in industrial scenarios with different sample distributions. This further confirms the effectiveness and generalizability of the proposed method, providing more comprehensive experimental support for its engineering applications.

### 4.6. Comparison Experiments

The primary engineering contribution of this research is that it overcomes the limitations of the “stable operating condition assumption” in industrial fault diagnosis. This advancement allows for accurate equipment monitoring under complex operating states that better reflect real-world conditions. The findings demonstrate high reliability and interpretability, which can improve the understanding of bearing fault diagnosis in practical industrial settings and support targeted maintenance actions when faults are identified.

Therefore, three comparative experiments were performed to further evaluate the performance advantages of the IBRB-a model proposed in this study. The first set compares it with the traditional IBRB model; the second group assesses the contribution of each core component of the IBRB-a model through ablation experiments; the third set involves comparisons with other methods in the field of deep learning. All methods were evaluated on the same dataset using consistent evaluation metrics, as in previous sections, to ensure objective and fair comparisons.

**Figure 17:**
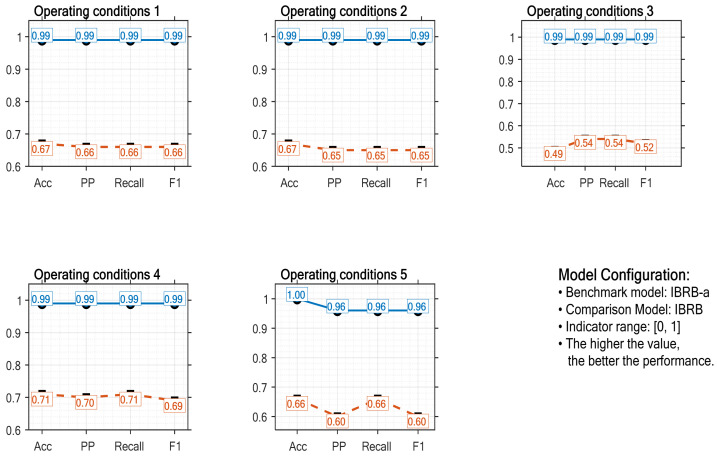
Line Charts of Four Indicators Under Different Conditions.

(1) Comparative experiment with the traditional IBRB model: As a classical framework for interval-valued belief rule-based models, the traditional IBRB model has been widely validated in bearing fault diagnosis. In this experiment, the IBRB-a model was compared with the traditional IBRB model across multiple performance dimensions. Both models used the same rule initialization strategy and dataset partitioning ratio. To mitigate random errors, 10 independent repeated trials were conducted. According to the statistical results presented in Figure 17 (The blue curve represents the IBRB-a model), the IBRB-a model achieves significant improvement across all four core metrics: it attains an accuracy rate of over 98% under each operating condition, precision and F1 score both exceed 95%, and the recall rate remains above 96%. These values reflect an increase in more than 28 percentage points compared to the traditional IBRB model. The results demonstrate that, while retaining the advantages of the traditional IBRB framework, the IBRB-a model achieves comprehensive performance enhancement through structural improvements.

(2) To quantitatively evaluate the contribution of each core component in the IBRB-a framework, a systematic ablation experiment is designed herein. This experiment compares four ablation model variants, as introduced in Table 17, where each variant removes one core component, and all experiments are conducted under the same 10-fold cross-validation framework.

The systematic ablation experiment reveals the differential contributions of each core component to the performance of the IBRB-a model, with specific data comparisons shown in Table 18.

(3) To confirm the competitiveness of the IBRB-a model, this experiment selects five mainstream methods: Support Vector Machine (SVM), K-Nearest Neighbors (KNN), Random Forest (RF), probabilistic spiking response model (PSRM) and Physics Guided Convolutional Neural Network (PGCNN). Where SVM, KNN and RF are traditional machine learning methods, PSRM is a multilayer probabilistic spike response model [48], and PGCNN uses a rectangular input structure and rectangular convolution kernels guided by physical principles [49].

**Figure 18:**
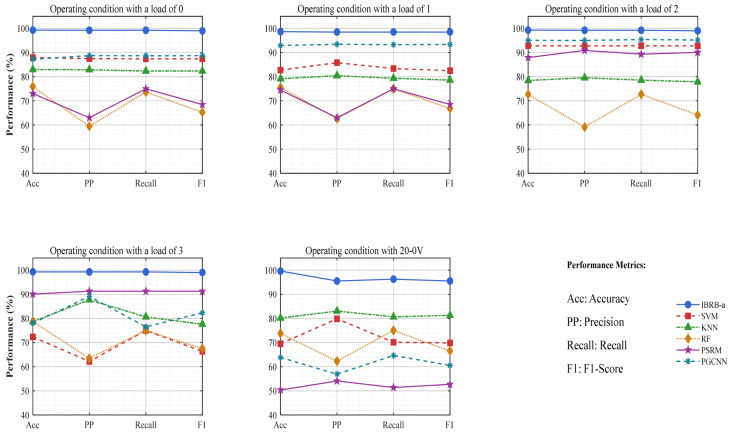
Comparison of the performance of six models.

**Figure 19:**
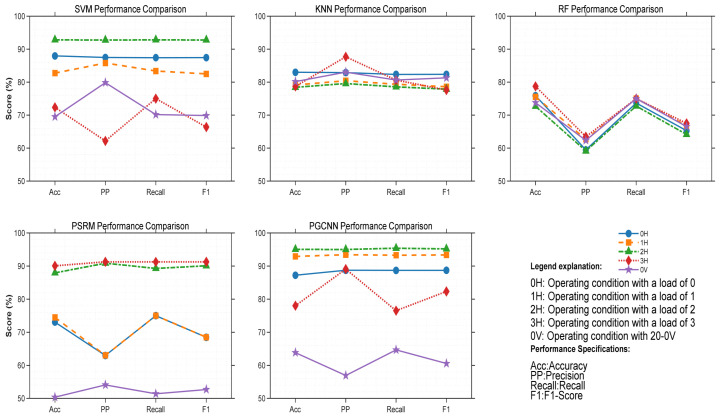
Comparative analysis of the performance of different models across different conditions.

As shown in the line chart in Figure 18 and the detailed results in Table 19, the IBRB-a model performs consistently well across different operating conditions in all four evaluation metrics significantly outperforming all other compared methods. Taking Operating Condition 1 as an example, the table data show that the IBRB-a model achieves an accuracy of 99.29%, precision of 99.25%, recall of 99.25%, and F1 score of 99.00%—these values are far higher than those of SVM (87.94%, 87.47%, 87.41%, 87.42%), KNN (82.98%, 82.80%, 82.33%, 82.35%), RF (75.89%, 59.46%, 73.65%, 65.26%), PSRM (73.05%, 62.97%, 75.00%, 68.46%), and PGCNN (87.23%, 88.72%, 88.69%, 88.71%). A comparative analysis can also be conducted from another perspective via the line chart in Figure 19 and the specific data in Table 19. By observing the indicator changes in various methods under different operating conditions, it is found that all indicators of the IBRB-a model remain at a high level across different operating conditions, with minimal fluctuations. In terms of accuracy, the IBRB-a model demonstrated highly consistent performance across five evaluation scenarios, with recorded values of 99.29%, 98.67%, 99.29%, 99.29%, and 99.57%. In contrast, the indicators of the other methods fluctuate significantly under different operating conditions. As an example, the SVM achieved accuracy rates of 87.94%, 82.73%, 92.81%, 72.34%, and 69.5% across the five test scenarios.

Under the scenario of bearing fault diagnosis with variable operating conditions, traditional machine learning methods are relatively sensitive to changes in operating conditions because of their inherent algorithmic characteristics, leading to relatively low performance across all evaluation indicators. Although deep learning methods such as the PSRM and PGCNN can handle complex data patterns to some extent, they often exhibit underperformance when operating conditions shift, negatively affecting their generalization capability. In contrast, the IBRB-a model benefits from its specific structure and optimization strategy, enabling effective feature extraction under varying conditions and stronger adaptability to operational shifts. This leads to its stability in all evaluated metrics.

This section systematically verifies the performance of the IBRB-a model through three sets of progressive experiments: benchmark model comparison, internal strategy verification, and cross-domain method benchmarking.

In Experiment 1, compared to the baseline IBRB model, IBRB-a achieved significant improvements of 35.07%, 35.27%, 33.93%, and 35.83% in average accuracy, precision, recall, and F1 score, respectively. This improvement effectively validates the effectiveness of the optimized rule generation and evidential reasoning processes within the IBRB-a model. Specifically, by introducing adaptive rule generation, the IBRB-a model mitigates the problems of rule combination explosion and conflict; its optimized inference mechanism directly enhances computational efficiency and decision reliability. This demonstrates that the proposed method successfully integrates the transparency of belief rule-based systems with the capability to model complex data patterns.

In Experiment 2, The P-CMA-ES constrained optimization exerts the most critical impact—its removal leads to a sharp drop of 16.15% in average accuracy, and the performance degradation even reaches 27.86% under the 3H high-load operating condition, which highlights the decisive role of physical constraints in guiding the optimization direction and preventing the model from falling into local optima under high-load and complex conditions. The KDE-based interval division is crucial for the model’s adaptability to operating conditions; particularly under the 0 V variable speed operating condition, its absence results in a 13.10% decrease in accuracy, verifying its core value in adaptively capturing changes in data distribution and resisting feature drift. In contrast, although the attention weight module and dynamic rule matching mechanism have relatively moderate direct impacts on the average performance (decreasing by 3.50% and 3.07% respectively), a deeper analysis reveals their indispensable role in enabling adaptive and interpretable reasoning. The attention module acts as the dynamic core that translates the model’s static knowledge into context-aware decisions. Under variable operating conditions, it selectively focuses on the most relevant and discriminative features for each specific input, effectively filtering out noise and irrelevant variations induced by load or speed changes. This capability is crucial for maintaining robustness when feature distributions shift. More importantly, the generated attention weights provide a transparent, sample-level explanation for the diagnostic process by quantitatively showing how much each feature contributed to activating each rule. This directly operationalizes the model’s interpretability during inference, making the decision logic traceable and trustworthy. They jointly constitute the basic robustness framework of the model, ensuring the focus on key features and flexible adjustment of inference strategies under different operating conditions. In summary, the IBRB-a model proposed in this study exhibits significant comprehensive advantages in bearing fault diagnosis under varying operating conditions. Its core strengths lie in the fact that the rationality and convergence efficiency of model parameter learning are ensured through P-CMA-ES optimization integrated with physical constraints, and strong adaptability to changes in data distribution under complex operating conditions is endowed to the model via KDE-based adaptive interval division. Meanwhile, intelligent focus on discriminative features and dynamic adjustment of the inference process are achieved by the model through embedding the attention mechanism and dynamic rule matching. This series of innovative designs enables the IBRB-a to not only achieve high accuracy but also maintain high interpretability, thereby successfully resolving the inherent contradiction between high-performance “black-box” models and low-performance interpretable models. Consequently, the IBRB-a provides a highly reliable, adaptable, and decision-transparent intelligent diagnosis solution for the industrial field, with important theoretical significance and engineering application value.

In Experiment 3, The IBRB-a model outperforms state-of-the-art models—including SVM, KNN, RF, PSRM, and PGCNN—across all four core metrics, with improvements ranging from 13% to 37%. (1) Compared with traditional machine learning models such as SVM and KNN, IBRB-a achieves an average performance gain of approximately 18%, highlighting its inherent advantages in handling complex nonlinear patterns and uncertain information. SVM is sensitive to kernel functions and parameter settings, while KNN is prone to local fluctuations under varying operating conditions. In contrast, the fuzzy rule-based inference framework of IBRB-a naturally accommodates uncertainty, making it more robust to changes in data distribution. (2) The superior performance of IBRB-a over more complex models such as RF and PGCNN is particularly noteworthy. Although RF possesses strong fitting capability, its black-box nature may lead to unstable generalization under varying operating conditions. PGCNN can capture spatiotemporal features but relies heavily on large-scale labeled data and lacks interpretability. The performance of IBRB-a demonstrates that in the context of fault diagnosis under varying operating conditions, hybrid-driven models that integrate domain knowledge with data are often more adaptive and reliable than purely data-driven models. (3) The most distinctive advantage of IBRB-a lies in the unified achievement of interpretability and stability. All comparative models sacrifice interpretability to varying degrees to improve performance, whereas IBRB-a achieves excellent diagnostic performance while maintaining high interpretability.

In summary, the experimental analysis quantitatively validates the superiority of the IBRB-a model and elucidates the sources of its advantages from a mechanistic perspective. Through its innovative adaptive rule learning and optimization architecture, the IBRB-a model successfully addresses the efficiency and conflict issues of traditional belief rule-based systems in high-dimensional and complex scenarios, while circumventing the common limitations of mainstream data-driven models, such as sensitivity to data quality and poor interpretability. The high performance, stability, and interpretability demonstrated by the IBRB-a model endow it with unique engineering application value in the field of bearing fault diagnosis under varying operating conditions, where reliable and transparent decision-making is required.

### 4.7. Analysis of Algorithm Performance

To comprehensively evaluate the efficiency of the IBRB-a model framework, this section provides an in-depth analysis from two perspectives: theoretical complexity analysis and practical runtime performance.

(1) Complexity Analysis: The time complexity of the IBRB-a model is primarily correlated with the dimension of the optimization problem W, the population size B, and the number of iterations Q. Let L denote the size of the rule base, N the number of belief degrees per rule, and M the number of attribute weights in the system. The time complexity per generation can be expressed as $O_{generation}\;=O(B\times E_{single}\;+B\times W^{2}+B\times W)$, where W satisfies the constraint W=L×N+L+M, and the complexity of each individual evaluation is $O(E_{single}\;)$. For large values of W, the computational cost is dominated by $O(B\times W^{2})$. Consequently, the total time complexity of the entire optimization process is $O_{total\;}=Q\times O_{generation}\;$. The spatial complexity is mainly determined by the storage requirements for the covariance matrix, the population, and auxiliary variables, which can be expressed as $O_{space}\;=O(W^{2}+B\times W)$.

(2) Practical Runtime Statistics. Taking the 0 Hz operating condition as an example, the actual performance of the algorithm in the experimental environment (MATLAB R2024a) is summarized in Table 20. The time allocation analysis indicates that the computational load is relatively balanced between the two optimization stages. This validates the rationality of the hybrid framework design, effectively avoiding the bottleneck effect inherent in single-optimizer approaches.

The proposed optimization framework exhibits favorable scalability in its theoretical design. Its time complexity is dominated by $O(Q\times B\times W^{2})$, and the space complexity is $O(W^{2}+B\times W)$, enabling it to handle computational demands of medium to large-scale problems. In terms of practical performance, through core designs such as the “global exploration-local tuning” two-stage synergy and domain knowledge-guided search, the algorithm achieves an effective balance between runtime efficiency and optimization accuracy. Experimental results demonstrate that the framework can generate high-precision, reliable, and structurally reasonable parameters for the belief rule base within a controllable timeframe. This verifies its practical value in complex system modeling and optimization tasks. These characteristics render the framework particularly suitable for complex optimization problems that demand high precision, strong constraints, and a certain degree of interpretability.

### 4.8. KDE Parameter Sensitivity Analysis

Prior to dynamic interval partitioning using KDE, a sensitivity analysis of KDE parameters was conducted to determine appropriate kernel functions and bandwidths.

(1) Kernel Function Sensitivity: Under a fixed bandwidth (determined by Silverman’s rule), four commonly used kernel functions were compared: Gaussian, Epanechnikov, triangular, and cosine. Table 21 presents the average diagnostic accuracy of the IBRB-a model under different kernel functions. As indicated in the table, the variations in average accuracy across different kernels are minimal. This suggests that model performance is insensitive to kernel selection when a reasonable bandwidth is applied. The Gaussian kernel was selected as the default due to its stable and marginally superior performance.

(2) Bandwidth Sensitivity: With the Gaussian kernel fixed, model performance was tested as the bandwidth coefficient ƛ varied within the range [0.7, 1.5]. Experimental results demonstrate that the model maintains an average accuracy plateau above 95% with a fluctuation range of less than ±1.21% as α varies across this wide interval. The performance curve exhibits a distinct “flat region,” verifying that the selected parameters lie within a robust zone. A significant performance degradation is only observed when the bandwidth is excessively small (ƛ < 0.5, leading to overfitting noise) or excessively large (ƛ > 1.8, resulting in the loss of feature details).

(3) Stability of Interval Reference Points: Beyond accuracy, the stability of the generated interval reference points under different KDE parameter settings was evaluated. Table 22 displays the interval partitioning results for feature F1 when the bandwidth coefficient α is set to 0.7, 1.0, and 1.3, respectively. As shown in Table 22, within the reasonable bandwidth range, the average offset rate of interval centers is less than 2%, indicating good stability of the KDE-based interval partitioning.

The aforementioned analysis indicates that the proposed KDE interval partitioning method offers threefold guarantees regarding parameter selection: a solid theoretical foundation; strong parameter robustness; and good structural fault tolerance. Consequently, the proposed method does not require tedious parameter tuning in practical engineering applications, demonstrating favorable utility and generalizability.

## 5. Conclusions

Based on the IBRB-r model, this study proposes a novel model named IBRB-a for bearing fault diagnosis under variable operating conditions. The model is designed to assist decision-makers in conducting clear, convenient, and efficient bearing fault prediction in practical industrial environments.

The IBRB-a model demonstrates practical utility in bearing fault diagnosis under variable operating conditions. It exhibits stable and superior diagnostic performance compared to the traditional IBRB model, as well as a range of machine learning and deep learning methods. The model is capable of adapting to dynamic industrial operating scenarios. It maintains interpretability through expert knowledge constraints, thereby avoiding the “black-box” problem, and enhances algorithm robustness via the introduction of random perturbation factors. This enables it to meet core industrial requirements for both reliability and interpretability in fault diagnosis. However, this study also objectively acknowledges the current limitations of the model: the adaptive capability of the model depends on the range of operating conditions covered by the training data, and its performance guarantee for completely unknown or extremely abrupt operating condition patterns requires further verification; the initialization of the model requires a certain amount of domain expert knowledge, which may affect its ability for rapid deployment in scenarios involving entirely new equipment or knowledge scarcity; despite the introduction of robustness designs, the foundation for rule matching and inference may still be challenged when facing extreme class imbalance or low-quality data.

Accordingly, future work will be deepened along the following paths: strategies combining meta-learning or online learning will be investigated to enable the model to achieve rapid self-update using a small number of new samples, thereby breaking through preset operating condition boundaries; methods integrating unsupervised feature learning with automated rule generation will be explored to reduce the model’s reliance on refined prior knowledge during construction; uncertainty quantification modules from evidence theory or hierarchical attention mechanisms will be introduced to enable the model not only to make diagnoses but also to evaluate the confidence of the diagnostic results, thereby making better decisions in the face of complex data.

This study not only proposes an effective bearing fault diagnosis model under varying operating conditions but also provides a theoretical basis and practical paradigm for constructing the next generation of data-intelligence integrated industrial health management systems.

## Figures and Tables

**Table 1:** Criteria for the interpretability of models.

Category	Guideline Description
Modeling process	Prerequisite attributes and completeness of diagnostic results
	Rationalization of the division of the reference interval
	Rationalization of the combination of interval rules
	The model parameters have actual physical significance
	The spacing rule base should maintain simplicity
Reasoning process	Reasonable information conversion of input and output data
	Interpretability of model reasoning
Optimization process	Reasonableness of the belief distribution
	Rational use of expert knowledge
	Parameter optimization with interpretable constraints

**Table 2:** Output reference value settings for fault types.

Reference Point	N	R	IR	OR
Reference value	0	1	2	3

**Table 3:** Experimental data and settings.

Group Category	Sample Type	Load Type	Normal	Ball	Inner Ring	Outer Ring
A	Train	0H	82	86	81	82
	Test	0H	36	32	37	36
B	Train	1H	84	88	85	74
	Test	1H	34	30	33	44
C	Train	2H	83	83	85	81
	Test	2H	34	35	33	37
D	Train	3H	81	79	88	83
	Test	3H	37	39	30	35

**Table 4:** Experimental data and settings.

Group Category	Sample Type	Load Type	Normal	Ball	Inner Ring	Outer Ring
E	Train	0 V	79	76	93	84
	Test	0 V	37	42	25	32

**Table 5:** Correlation coefficients.

	0H	1H	2H	3H
Mean	0.9257	0.9269	0.9238	0.9144
Peak	0.9450	0.9421	0.9336	0.9292
RMS	1.0000	1.0000	1.0000	1.0000
Kurtosis	0.8427	0.8642	0.8662	0.8729
Skewness	0.6885	−0.6843	−0.7606	−0.7150
Waveform Factor	0.9401	0.9513	0.9561	0.9492
Impulse Factor	0.9374	0.9575	0.9680	0.9715
Margin Factor	0.5096	0.6521	0.6152	0.4013

**Table 6:** Initial rules of the IBRB-a.

Number	Referential Interval	Rule Reliability	The Rule Reliability Constraint	Rule Weight	The Rule Weight Constraint	The Initial Belief
1	[0.28, 0.42]	1	0.5–1	1	0.5–1	{0.05, 0.20, 0.70, 0.05}
2	[0.42, 1.10]	1	0.5–1	1	0.5–1	{0.08, 0.17, 0.68, 0.07}
3	[1.10, 1.34]	1	0.5–1	1	0.5–1	{0.03, 0.15, 0.72, 0.10}
4	[1.34, 1.44]	1	0.5–1	1	0.5–1	{0.06, 0.18, 0.69, 0.07}
5	[1.44, 1.60]	1	0.5–1	1	0.5–1	{0.02, 0.16, 0.73, 0.09}
6	[1.60, 2.95]	1	0.5–1	1	0.5–1	{0.04, 0.19, 0.67, 0.10}
7	[2.95, 3.17]	1	0.5–1	1	0.5–1	{0.07, 0.14, 0.70, 0.09}
…	…	…	…	…	…	…
40	[8.42, 9.00]	1	0.5–1	1	0.5–1	{0.07, 0.15, 0.68, 0.10}

**Table 7:** CAM-ES optimization model parameters at 0H.

Number	Rule Reliability	Rules Weights	Output Results
1	0.3354	0.8353	{0.40, 0.08, 0.38, 0.14}
2	0.2192	0.4854	{0.29, 0.34, 0.26, 0.11}
3	0.8172	0.0001	{0.34, 0.01, 0.40, 0.24}
4	0.0798	0.0001	{0.33, 0.11, 0.28, 0.28}
5	0.4160	0.0644	{0.16, 0.18, 0.16, 0.50}
6	0.3148	0.0001	{0.32, 0.33, 0.13, 0.21}
7	0.0611	0.0036	{0.36, 0.03, 0.22, 0.39}
8	0.2013	0.0008	{0.26, 0.25, 0.26, 0.23}
…	…	…	…
40	0.3515	0.4758	{0.16, 0.18, 0.16, 0.50}

**Table 8:** P-CAM-ES Optimization Model Parameter Table of 0H.

Number	Rule Reliability	Rules Weights	Output Results
1	0.8616	0.4241	{0.59, 0.09, 0.00, 0.35}
2	0.5156	0.6343	{0.00, 0.34, 0.00, 0.75}
3	0.5794	0.4016	{0.59, 0.29, 0.00, 12.0}
4	0.9313	0.5435	{0.50, 0.00, 0.49, 0.00}
5	0.9231	0.1531	{0.86, 0.00, 0.15, 0.00}
6	0.8577	0.0001	{0.00, 0.00, 0.24, 0.78}
7	0.7467	0.0001	{0.37, 0.13, 0.13, 0.37}
8	0.8379	0.0002	{0.00, 0.00, 0.75, 0.24}
…	…	…	…
40	0.9907	0.3947	{0.22, 0.05, 0.74, 0.00}

**Table 9:** P-CAM-ES Optimization Algorithm Parameter Settings of 0H.

The Parameter	Value of Initial Parameter
Iteration number Q	700
Step size v	0.2
European distance size d	2
disturbance coefficient ϑ	Random number

**Table 10:** Fault Diagnosis Performance of 0H IBRB-a.

Result	G_1_	G_2_	G_3_	G_4_	TP + FP	PP
G_1_	36	0	0	0	36	1
G_2_	0	32	0	0	32	1
G_3_	0	1	36	0	37	0.97
G_4_	0	0	0	36	36	1
TP + FN	36	33	36	36	141	/
Recall	1	0.97	1	1	/	/
F1	1	0.98	0.98	1	/	/

**Table 11:** Fault Diagnosis Performance of 1H IBRB-a.

Result	G_1_	G_2_	G_3_	G_4_	TP + FP	PP
G_1_	32	2	0	0	34	0.94
G_2_	0	30	0	0	30	1
G_3_	0	0	33	0	33	1
G_4_	0	0	0	44	44	1
TP + FN	32	32	33	44	141	/
Recall	1	0.94	1	1	/	/
F1	0.97	0.97	1	1	/	/

**Table 12:** Fault Diagnosis Performance of 2H IBRB-a.

Result	G_1_	G_2_	G_3_	G_4_	TP + FP	PP
G_1_	34	0	0	0	34	1
G_2_	0	35	0	0	35	1
G_3_	0	0	33	0	33	1
G_4_	0	0	1	36	37	0.97
TP + FN	34	35	34	36	139	/
Recall	1	1	0.97	1	/	/
F1	1	1	0.98	0.98	/	/

**Table 13:** Fault Diagnosis Performance of 3H IBRB-a.

Result	G_1_	G_2_	G_3_	G_4_	TP + FP	PP
G_1_	36	1	0	0	37	0.97
G_2_	0	39	0	0	39	1
G_3_	0	0	30	0	30	1
G_4_	0	0	0	35	35	1
TP + FN	36	40	30	35	141	/
Recall	1	0.97	1	1	/	/
F1	0.98	0.98	1	1	/	/

**Table 14:** CAM-ES optimization model parameters at 0 V.

Number	Rule Reliability	Rules Weights	Output Results
1	0.2242	0.8743	{0.49, 0.02, 0.00, 0.48}
2	0.6409	0.1979	{0.63, 0.02, 0.01, 0.34}
3	0.5521	0.0614	{0.30, 0.23, 0.04, 0.43}
4	0.7669	0.0001	{0.23, 0.32, 0.03, 0.43}
5	0.0091	0.0001	{0.15, 0.25, 0.04, 0.56}
6	0.0441	0.0217	{0.03, 0.09, 0.09, 0.79}
7	0.7996	0.5551	{0.18, 0.02, 0.00, 0.80}
8	0.3781	0.9083	{0.00, 0.03, 0.01, 0.96}
…	…	…	…
40	0.6120	0.4547	{0.03, 0.33, 0.32, 0.33}

**Table 15:** P-CAM-ES optimization model parameters at 0 V.

Number	Rule Reliability	Rules Weights	Output Results
1	0.7385	0.8211	{0.05, 0.16, 0.01, 0.78}
2	0.7414	0.7488	{0.10, 0.39, 0.01, 0.50}
3	0.8910	0.8328	{0.10, 0.01, 0.01, 0.89}
4	0.5727	0.8642	{0.09, 0.04, 0.00, 0.86}
5	0.6537	0.2927	{0.33, 0.00, 0.00, 0.67}
6	0.9035	0.5109	{0.13, 0.01, 0.00, 0.85}
7	0.6372	0.1740	{0.13, 0.06, 0.00, 0.82}
…	…	…	…
40	0.6607	0.6225	{0.07, 0.06, 0.74, 0.13}

**Table 16:** Fault diagnosis performance of 0 V IBRB-a.

Result	G1	G2	G3	G4	TP + FP	PP
G1	33	4	0	0	37	0.89
G2	0	42	0	0	42	1
G3	0	1	24	0	25	0.96
G4	0	0	1	31	32	0.97
TP + FN	33	47	25	31	136	/
Recall	1	0.89	0.96	1	/	/
F1	0.94	0.94	0.96	0.98	/	/

**Table 17:** Meaning of Symbols for Ablation Model Variants.

Model Symbol	Meaning
IBRB-a	Full model
IBRB-a-K	Remove KDE-based binning and use equal-width binning instead
IBRB-a-D	Remove the dynamic rule matching mechanism and use static rule matching
IBRB-a-A	Remove the attention weight module and use fixed weight allocation
IBRB-a-P	Remove P-CMA-ES constrained optimization and use the original P-CMA-ES

**Table 18:** Performance Comparison of Ablation Experiment Models Under Different Working Conditions.

Group	Method	Acc (%)	PP (%)	Recall (%)	F1 (%)
A	IBRB-a	99.29	99.25	99.25	99.00
	IBRB-a-K	91.23	90.41	91.23	90.41
	IBRB-a-D	94.33	93.40	95.32	94.22
	IBRB-a-A	93.62	93.46	93.08	93.25
	IBRB-a-P	91.23	92.13	98.02	94.05
B	IBRB-a	98.67	98.50	98.50	98.50
	IBRB-a-K	94.32	95.46	94.32	94.32
	IBRB-a-D	92.20	92.18	92.20	92.08
	IBRB-a-A	92.91	92.16	93.14	92.60
	IBRB-a-P	88.69	88.69	90.10	88.41
C	IBRB-a	99.29	99.25	99.25	99.00
	IBRB-a-K	96.31	96.31	96.30	96.32
	IBRB-a-D	90.07	88.76	88.97	88.86
	IBRB-a-A	91.49	88.56	89.16	88.84
	IBRB-a-P	79.43	79.85	83.12	79.85
D	IBRB-a	99.29	99.25	99.25	99.00
	IBRB-a-K	92.13	93.45	94.12	92.13
	IBRB-a-D	89.36	82.48	82.74	82.58
	IBRB-a-A	90.78	90.84	91.88	91.30
	IBRB-a-P	72.43	73.41	81.06	86.34
E	IBRB-a	99.59	95.50	96.25	95.50
	IBRB-a-K	86.49	86.58	92.13	86.58
	IBRB-a-D	94.33	94.46	93.75	93.94
	IBRB-a-A	93.62	93.46	93.08	93.25
	IBRB-a-P	83.21	84.76	91.06	84.23

**Table 19:** Comparison of the performance of six models under different conditions.

Group	Method	Acc (%)	PP (%)	Recall (%)	F1 (%)
A	IBRB-a	99.29	99.25	99.25	99.00
	SVM	87.94	87.47	87.41	87.42
	KNN	82.98	82.80	82.33	82.35
	RF	75.89	59.46	73.65	65.26
	PSRM	73.05	62.97	75.00	68.46
	PGCNN	87.23	88.72	88.69	88.71
B	IBRB-a	98.67	98.50	98.50	98.50
	SVM	82.73	85.79	83.37	82.49
	KNN	79.14	80.41	79.35	78.58
	RF	75.54	62.68	75.00	66.83
	PSRM	74.47	63.00	75.00	68.48
	PGCNN	92.91	93.43	93.27	93.35
C	IBRB-a	99.29	99.25	99.25	99.00
	SVM	92.81	92.75	92.81	92.76
	KNN	78.42	79.56	78.56	77.87
	RF	72.66	59.12	72.73	64.13
	PSRM	87.94	90.86	89.29	90.07
	PGCNN	95.04	95.00	95.39	95.19
D	IBRB-a	99.29	99.25	99.25	99.00
	SVM	72.34	62.17	75.00	66.37
	KNN	78.72	87.67	80.62	77.60
	RF	78.72	63.46	75.00	67.50
	PSRM	90.07	91.28	91.25	91.26
	PGCNN	78.01	89.08	76.52	82.32
E	IBRB-a	99.59	95.50	96.25	95.50
	SVM	69.50	79.84	70.14	69.85
	KNN	80.14	83.03	80.64	81.28
	RF	73.76	62.32	75.00	66.51
	PSRM	50.35	54.07	51.39	52.69
	PGCNN	63.83	56.92	64.64	60.54

**Table 20:** Algorithm Runtime Performance Statistics.

Performance Indicators	Numerical Value	Analysis Explanation
Total runtime	77.44 s	Meet real-time requirements
CMA-ES Phase Time	39.03 s (50.4%)	Mainly responsible for global search
PC-MAES Phase Time	38.26 s (49.4%)	Mainly responsible for local fine-tuning
Total function call count	1.94 × 10^7^ times	Includes internal ER calls
Final MSE	3.965 × 10^−3^	High prediction accuracy
Classification Accuracy	0.9929	Meet practical application requirements

**Table 21:** Comparison of Model Performance Under Different Kernel Functions (Accuracy, %).

Kernel Function	0H	1H	2H	3H	0 V	Average
Gaussian kernel	99.29	98.67	99.29	99.29	99.59	99.23
Epanechnikov kernel	97.16	95.04	98.58	96.45	97.16	96.88
Triangular	97.87	95.74	97.87	96.45	97.16	97.01
Cosine Kernel	96.45	92.20	95.74	95.03	95.74	95.03

**Table 22:** Comparison of Interval Centers Under Different Bandwidths.

Interval Number	ƛ = 0.7	ƛ = 1.0	ƛ = 1.3	Average Offset Rate
I_1_	0.215	0.223	0.220	1.82%
I_2_	0.634	0.623	0.628	0.89%
I_3_	1.052	1.045	1.048	0.33%
I_4_	1.673	1.670	1.668	0.15%
I_5_	2.285	2.293	2.278	0.33%
I_6_	2.847	2.852	2.839	0.23%
I_7_	3.124	3.118	3.121	0.10%
I_8_	3.672	3.665	3.669	0.09%
I_9_	4.223	4.231	4.218	0.15%

## Data Availability

The data used in this study are publicly available from the following sources: (1) The Case Western Reserve University (CWRU) bearing dataset is available at https://gitcode.com/open-source-toolkit/d1ef9, accessed on 3 July 2025. (2) The Southeast University (SEU) gearbox dataset is available at https://gitcode.com/Resource-Bundle-Collection/8994d, accessed on 28 July 2025.

## Appendix A

**Table A1:** Table of Formula Letter Meanings.

Initials	Connotation	Initials	Connotation
$\hat{H}$	the actual extracted fault feature	D	the total number of training sample data
H(x)	the nonlinear mapping	U	the model’s output value
η	non-stationary noise	$U^{*}$	the system’s label value
Ω	the effective region where the key fault features should lie	$\Psi^{0}$	the initial parameter set
a	the preset high-reliability threshold	$x_{k}^{(g+1)\;}$	the k-th individual in the g+1 generation
X	the input feature space	ϑ	disturbance coefficient
Y	the fault mode output space	$z_{k}$	a random vector
k	the initial operating condition identifier	u	the number of additional individuals
$k^{\prime }$	the new operating condition identifier	$\xi^{\left(S\right)}$	the population of the g generation
$P_{k}$	the initial rule	EXP	expert knowledge
$Dom\;\left(P_{k}\right)$	the effective domain of $P_{k}$	${eq}_{mean}$	the mean value of the current population
Ψ	the optimization parameter	$eq_{k}$	the newly generated individual
$\Psi_{*}(t)$	the optimal solution	d	the maximum allowable distance
t	time	$can_{k}$	the *k*-th candidate solution vector
$\hat{\Psi }(t)$	the parameter obtained by the algorithm	$can_{0}$	the initial population vector
R	the correlation degree between the parameters and diagnostic results	K	the index set of inactive rules
ε	the acceptable threshold	$I_{k}$	the index range
τ	the interpretability threshold	$\omega_{k}$	the weight of the *k*-th attribute
${\hat{g}}_{s}(y)$	the probability density estimation function of the s-th input feature	$\omega_{k,\min}$	the minimum threshold of the attribute weights
num	the total number of samples	$\beta_{k}$	the confidence value
$w_{s}$	the bandwidth of the s-th feature	$\beta_{0}$	the initial confidence value
L(•)	the kernel function	v	the maximum threshold for confidence variation
$y_{k,s}$	the value of the *k*-th sample on the *s*-th feature	$A_{eq}$	the equality constraint matrix
$\phi_{i}$	the matching degree	$b_{eq}$	the right-hand side vector
mod	the center of the reference interval,	$\vec{x}$	the solution vector
$\left[p_{i},q_{i}\right]$	the interval boundary	$b_{i}$	the target value of the i-th constraint
$x_{i}$	sample data	$n_{j}$	the number of nonzero components
$\gamma_{i}$	the activation weight	∂	the sum of deviations
γ	the rule weight	$\xi_{i:\lambda }^{\left(g\right)}$	the i-th optimal individual
$\gamma_{\min}$	the minimum activation weight	$\varpi_{i}$	the exponential decay weight
Θ	the frame of discernment	μ	the number of preferred individuals
$G_{n}$	the evaluation grades for bearing fault diagnosis results	λ	the number of offspring
N	the number of evaluation grades	C	the covariance matrix
$\beta_{n,k}$	the $G_{n}$ confidence level of the result	$c_{1}$	the rank-one update learning rate
$\beta_{\Theta ,k}$	The *k*-th attribute in the frame of discernment is globally unknown	$c_{\mu }$	the rank −μ update learning rate
$b_{rw,k}$	the normalization factor	$path_{c}$	the evolutionary path of the covariance matrix
$m_{n,k}$	the probability mass of the *k*-th piece of evidence at the specific evaluation grade $G_{n}$	$h_{\sigma }$	the heuristic indicator
$\beta_{G,\delta (k)}$	the joint support degree of S independent pieces of evidence	$Z_{i}$	the accumulation coefficient
$\delta_{k}$	the joint probability mass	$\alpha_{i}$	the attention weight
Z	the final utility value	${\vec{x}}_{i:\lambda }^{\left(g\right)}$	candidate solution
$\sigma (G_{n})$	the utility score of $G_{n}$	$m^{\left(g\right)}$	the mean vector
$TP_{i}$	the number of true positives	$step^{\left(g\right)}$	the step size of the g-th generation
$FP_{i}$	the count of true positives	ACC	accuracy
$FN_{i}$	the number of false positives	PP	the positive predictive value
$TN_{i}$	the count of true negatives	Q	Iteration number

**Algorithm A1.** Pseudocode content	
Stage	Pseudocode
Initialization Phase	1: Load training data D_train and testing data D_test 2: Normalize input features if necessary 3: Initialize belief degree matrix β = β_init 4: Initialize rule weights r = [1, 1, …, 1] ∈ ℝ^L 5: Initialize attribute weights w = [1, 1, …, 1] ∈ ℝ^L 6: Define assessment grades D = {D_1, D_2, D_3, D_4} 7: Define constraint matrices A_eq, b_eq for parameter optimization
Parameter Optimization using CMA-ES	8: Define optimization vector: θ = [vec(β), r, w] ∈ ℝ^{L × N + L + L} 9: Set lower bounds lb = 0.0001, upper bounds ub = 1 10: for generation = 1 to G do 11: Generate population of λ candidate solutions: 12: θ_k = θ_mean + σ × B × (D ⊙ N(0,1)), for k = 1, …, λ 13: 14: Apply constraints to each candidate: 15: - Projection to satisfy A_eq × θ_k = b_eq 16: - Confidence degree constraints (sum to 1 per rule) 17: - Monotonicity constraints for belief degrees 18: - Rule weight constraints (r_k ≥ 0.5) 19: - Attribute weight constraints (w_k ≥ 0.4) 20: 21: Evaluate fitness for each candidate: 22: for k = 1 to λ do 23: MSE_k = CalculateMSE(θ_k, D_train) ▷ Using Algorithm A2 24: end for 25: 26: Select best μ candidates based on fitness 27: Update θ_mean using weighted recombination 28: Update evolution paths p_c, p_s 29: Update covariance matrix C 30: Update step size σ 31: end for 32: θ^* = best candidate from final generation

**Algorithm A2.** Pseudocode content	
Stage	Pseudocode
Adaptive Rule Matching and Inference	1: Parse θ into β, r, w 2: Initialize MSE = 0 3: for each sample (x, y) in Dataset do 4: for each attribute m = 1 to M do 5: Find reference interval [a_{m,k}, a_{m,k + 1}] containing x_m 6: Calculate matching degrees: 7: α_{m,1} = (a_{m,k + 1} − x_m)/(a_{m,k + 1} − a_{m,k}) 8: α_{m,2} = 1 − α_{m,1} 9: 10: Retrieve belief degrees for adjacent rules: 11: β_{m,1} = belief degrees of rule k for attribute m 12: β_{m,2} = belief degrees of rule k + 1 for attribute m 13: 14: Fuse using analytical ER: 15: β_m = ER_Fusion([α_{m,1}, α_{m,2}], [β_{m,1}, β_{m,2}]) 16: end for 17: 18: Calculate activation weights: 19: for each rule l = 1 to L do 20: ω_l = (r_l × w_l)/(∑_{j = 1}^L r_j × w_j) 21: end for 22: 23: Aggregate beliefs using ER rule: 24: β_final = ER_Aggregation({(ω_l, β_l)}_{l = 1}^L) 25: 26: Calculate output: 27: ŷ = ∑_{n = 1}^N β_{final,n} × utility(D_n) 28: 29: Update MSE: MSE = MSE + (ŷ − y)^2 30: end for 31: return MSE/N_samples
Testing and Evaluation Phase	33: for each test sample (x, y) in D_test do 34: ŷ = BRB_Inference(x, θ^*) ▷ Using steps 4–27 35: Store prediction ŷ 36: Calculate prediction error 37: end for 38: 39: Calculate overall metrics: 40: MSE_test = (1/N_test) × ∑_{j = 1}^{N_test} (ŷ_j − y_j)^2 41: Acc = (Number of |ŷ_j − y_j| < threshold)/N_test 42: return θ^*, predictions, MSE_test, Acc
